# Diversity of antibiotic resistance genes increases in urbanized lakes: A multi-tool screening

**DOI:** 10.1016/j.isci.2026.115892

**Published:** 2026-04-27

**Authors:** Pau De Yebra, Luca Zoccarato, John A. Galindo, Daniela Numberger, Nafi’u Abdulkadir, Hans-Peter Grossart, Alex D. Greenwood

**Affiliations:** 1 Leibniz Institute for Zoo and Wildlife Research, Alfred-Kowalke-Strasse 17, 10315 Berlin, Germany; 2 Leibniz Institute of Freshwater Ecology and Inland Fisheries, Department, of Plankton and Microbial Ecology, Zur alten Fischerhütte 2, 16775 Stechlin, Germany; 3 University of Potsdam, Institute of Biochemistry and Biology, Maulbeerallee 2, 14469 Potsdam, Germany; 4 Institute of Computational Biology, University of Natural Resources and Life Sciences (BOKU), Muthgasse 18, Vienna 1190, Austria; 5 Core Facility Bioinformatics, University of Natural Resources and Life Sciences (BOKU), Muthgasse 18, Vienna 1190, Austria; 6 Sokoto State University, Department of Microbiology, Birnin Kebbi Rd, Sokoto 852101, Nigeria; 7 Berlin-Brandenburg Institute of Advanced Biodiversity Research (BBIB), Altensteinstrasse 32, 14195 Berlin, Germany; 8 Freie Universität Berlin, Department of Veterinary Medicine, Institute for Virology, Robert von Ostertag-Strasse 7-13, 14163 Berlin, Germany

**Keywords:** Genes, Microbial genetics, Biological sciences tools

## Abstract

Antimicrobial resistance (AMR) threatens to cause up to 10 million deaths annually by 2050 if no action is taken. Using shotgun sequencing, we examined the eco-evolutionary dynamics of AMR across urban-rural environment gradients in lake water and sediments, a farm pond and a wastewater treatment plant (WWTP). ARGs were identified using multiple databases and five bioinformatic tools, detecting up to 18 ARG classes—more than any single tool alone. ARG diversity was higher in urban lake sediments, urban waters, and wastewater compared to rural lake sediments and water. Among all environments, urban lake water showed the highest overall ARG abundance, second only to wastewater, and this pattern held across all ARG classes, except for aminoglycoside resistance, which was most prevalent in rural lake sediments. These findings highlight multi-tool ARG screening efficiency, WWTPs and urban sediments as major ARG reservoirs, and emphasize the need for enhanced urban-rural AMR surveillance.

## Highlights

• A multi-tool screening approach and ABRicate detected the most AMR classes
• After wastewater, urban lake surface water showed the highest overall ARG abundance
• Lake sediments are an important reservoir for AMR both urban and rural lake sediments
• Multiple ARG screening approaches are key for detecting ARGs in aquatic environments

## Graphical abstract

**Figure:**
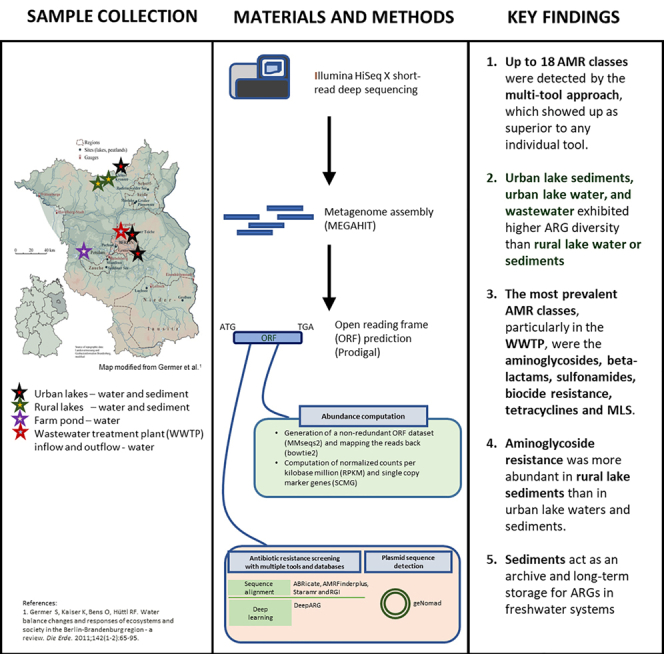

## Introduction

Despite increasing information on microbial communities in urban water environments,^1^^,^^2^^,^^3^^,^^4^ it is still uncertain whether similar patterns govern the abundance and diversity of antibiotic resistance genes (ARGs),^5^ as the impact of human activities can differ within the same biome.^6^^,^^7^^,^^8^ Urban water systems, such as lakes or rivers, have been described as reservoirs and major sources of ARGs from domestic, industrial, and hospital wastewater effluents.^9^^,^^10^^,^^11^^,^^12^^,^^13^ Urban lakes with high anthropogenic activity and industrial effluent exposure exhibit high concentrations of ARGs and heavy metals.^14^ Rural environments instead show diverse ARG profiles, linked to land use, agricultural practices, and environmental factors.^15^^,^^16^ Urban areas often have higher overall ARG loads but rural settings can harbor unique ARG profiles and high abundances in specific locations, such as agricultural areas or areas with proximity to animal husbandry or aquaculture.^17^ While urban and rural areas exhibit different patterns and drivers, they can be interconnected through various pathways, including wastewater discharge, animal movement, and human activity.

Understanding ARG profiles and distribution in aquatic environments is crucial because of the major threat that antimicrobial resistance (AMR) poses to environmental, animal, and human health.^18^ Recent evidence suggests that anthropogenic impact strongly influences the development, transmission, spread, and persistence of ARGs.^19^ AMR dynamics, particularly in urban aquatic environments exposed to activities such as wastewater treatment, aquaculture, clinical waste, or landfills, remain complex and poorly understood.^18^ This is particularly true for wastewater treatment plants (WWTPs), which can act as hotspots for ARGs in the aquatic environment.^20^^,^^21^

Advances in sequencing technologies and bioinformatics have improved ARG detection and classification in clinical and environmental samples, including the detection of mobile genetic elements (MGEs) such as plasmids, transposons, and proviruses integrated in host genomes, which can play a key role in AMR transmission.

Several studies have raised concerns about how different ARG detection tools and databases may not be suitable for detecting ARGs at low abundance (<1% of bacterial populations).^22^^,^^23^ Different tools vary in their ability to detect ARG classes due to differences in methodological approaches, databases, nomenclature, and capacity to detect point mutations. In that regard, many databases are biased toward clinical resistance genes or clinical isolates,^24^^,^^25^ introducing selective bias when annotating environmental ARGs. For instance, the comprehensive antibiotic resistance database (CARD) database^26^ includes a greater diversity of microbial genera and AMR variants than other databases, which could make it better suited for environmental screening of ARGs. Recent studies also suggest that each method is better suited for detecting specific classes of ARGs^27^ and most benchmarking has focused on isolates rather than complex environmental samples. Relying on a single tool or database often causes discrepancies in ARG profiling due to variations in annotation, structure, and database content.^24^ Comparative studies of these tools for samples containing mixtures or pollutants (soil, water, and sludge) remain scarce and can produce inconsistent results regarding ARG abundance and diversity. Urban areas typically show higher, diverse resistomes, while rural areas often show distinct, agriculture-related profiles. Key biases include sparse rural sampling and over-reliance on urban data.^28^^,^^29^ Indeed, rural areas are often underrepresented with only around 13%–19% of total studies and with rural monitoring more dependent on traditional culture-based methods, whereas urban studies use advanced sequencing, making direct, unbiased comparison difficult. Also, soil-based, agricultural (manure) AMR, common in rural areas can have varied, inconsistent resistance patterns compared to urban, wastewater-dominated environments. To this end, harmonized AMR output comparison tool ER (hAMRoaster) has been developed to assess the performance of different ARG tools using a synthetic dataset from strains isolated from human tissues.^22^ In a high-resistance mock community test, all tools exhibited high precision and accuracy, yet they still showed differences in ARG detection across ARG classes. The main limitation is that similar comparisons of ARG screening tools for application to environmental samples are entirely lacking,^30^ and predicting environmental ARGs based on a single ARG screening tool may miss some key ARG classes.

To better address the potential connectivity across urban and rural freshwater ecosystems, WWTPs and the knowledge gaps existing in characterizing ARG abundance and diversity, we performed shotgun metagenomic sequencing on water and sediment samples collected in the German states of Berlin and Brandenburg. We adopted an ensemble approach, combining 5 ARG screening tools and 9 ARG databases, to overcome the bioinformatic limitations described previously. We aim to investigate differences in removal efficiencies among WWTPs regarding ARGs and ARG classes. We use a multi-tool screening approach, which better detects diverse and abundant ARG classes. Identifying differences in ARG profiles and diversity among WWTP in- and outflow, and lake environments will help to understand ARG dynamics and improve protocols to improve WWTP removal efficiency without biases.

## Results

### AMR class detection

**Figure 1:**
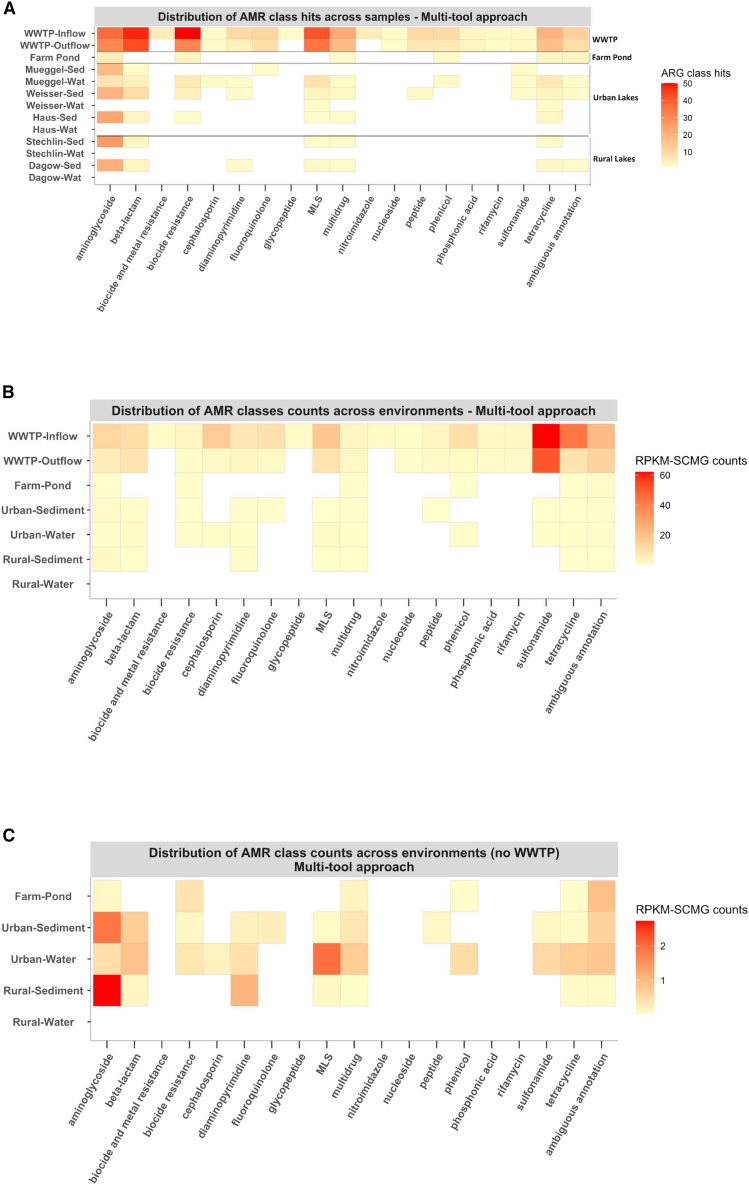
Distribution of antibiotic resistance genes (ARGs) hits and counts predicted in each sample or environmental group and classified by the antibiotic resistance class Open reading frames (ORFs) were annotated for antibiotic resistance genes (ARGs) and assigned to an AMR class. Each square in the heatmap corresponds to (A) the total number of ARG sequences per sample or (B and C) the mean rank of the reads per kilobase per million mapped reads normalized by single copy marker genes (RPKM-SCMG) linked to an AMR class for each environment, showing (B) or hiding (C) the WWTP. MLS, macrolides-lincosamides-streptogramins.

Up to 18 ARG classes were detected using the multi-tool approach (Figure 1A), with the cutoff set to antibiotic resistance genes (ARGs) detected by at least 2 tools. Diversity of ARG classes was found in descending order from WWTP inflow (all 18 classes) > WWTP outflow (16 classes) > Müggelsee water (10 classes) > Weisser See sediment (9 classes) > Dagowsee sediment (6 classes) = Stechlinsee sediment (6 classes) = Haussee sediment (6 classes) = farm pond (6 classes) > Müggelsee sediment (4 classes) > Weisser See water (2 classes), and no ARG hits were detected in the surface water of Haussee, Stechlinsee, or Dagowsee. Considering environmental groups (Figures 1B and 1C), the WWTP in- and outflow accounted for the highest number of classes, followed by urban sediments and urban water (both with 10 classes, which included lakes Haussee, Müggelsee, and lake Weisser See), rural sediments (6 classes, which included lakes Dagowsee and Stechlinsee), and farm pond (6 classes each). No ARG classes were detected in the rural surface waters. We could detect up to 511 ARG hits (from a total of 730 hits) in the WWTP in- and outflow, with the most prevalent drug classes being aminoglycosides (37 hits in the inflow and 31 hits in the outflow), beta-lactams (48 hits in the inflow and 43 hits in the outflow), biocide resistance (50 hits in the inflow and 31 hits in the outflow) and macrolides-lincosamides-streptogramins (MLS) (42 hits in the inflow and 35 hits in the outflow). A total of 162 ARG hits were predicted in the urban and rural sediments, with the majority of ARG classes associated with aminoglycosides.

In the current study, aminoglycosides, beta-lactams, sulfonamides, phenicols, macrolides, lincosamides, and peptides were identified in urban lakes, either in water, sediments or both. Aminoglycosides, beta-lactams, MLS, and tetracyclines were also identified in the rural sediments. In contrast, no rifamycin was detected in farm ponds, urban or rural lakes, or in water or sediments. We detected fluoroquinolone ARGs only in WWTP in- and outflow, and in urban sediments. In contrast, tetracycline ARGs were detected in all environments, except rural surface water. Nearly 69% of all ARGs were unique to WWTP in- and outflow, compared with only 4.1% of the bacterial taxa. Urban water and WWTP shared 16% of the identified ARGs, compared with 3.9% of shared bacterial taxa. These two groups alone comprised 85% of the overlapping ARGs but only approximately 8% of the shared bacterial taxa.

Both WWTP in- and outflow had the highest AMR normalized counts (reads per kilobase million by single-copy marker genes, RPKM-SCMG) when compared to all other studied environments. The highest abundance was detected in the ARG classes of sulfonamides (in the WWTP in- and outflow), and tetracyclines (in the WWTP inflow) (Figure 1B). Other classes that showed high abundance in the WWTP in- and/or outflow were the aminoglycosides, beta-lactams, cephalosporins, fluoroquinolones, MLS, and phenicol. WWTP samples had much higher RPKM-SCMG values than all other samples; we subtracted them to highlight patterns in the other environments (Figure 1C). Aminoglycosides constitute the AMR class with the highest mean RPKM-SCMG across all freshwater environments, being more prevalent in rural sediment than in urban water. The results show that MLS is more abundant in urban water than in other environments, and that urban water shows the highest abundance across all detected ARG classes. Moreover, diaminopyrimidine is more abundant in rural sediments than in sediments and water from urban environments. We also observed that the farm pond shares most of the predicted ARG classes with urban water, followed by urban sediments. Our results also show that WWTP in- and outflow exhibit the highest ARG overlap, with 144 genes shared between the two. However, the WWTP inflow contained 70 unique ARGs, whereas the outflow contained only one (Figure 4).

A total of six ARG classes were detected, instead, in the sediment samples from rural lakes Dagowsee and Stechlinsee, sharing up to six drug classes between the two lakes and differing in two drug classes and diaminopyrimidine (only detected in Dagowsee) (Figure 1A). A higher number of aminoglycoside hits were detected in urban sediments (61 ARG hits) compared to the rural sediments (47 ARG hits). Urban and rural lake sediments showed a similar number of aminoglycoside hits, whereas water samples from Lake Müggelsee showed the highest number of ARG hits among the freshwater lakes. The surface water of urban lakes Weisser See and Müggelsee differed in the number of ARG classes, with up to 10 in Müggelsee and only 2 in Weisser See (MLS and tetracycline, both shared with Lake Müggelsee).

### Detection profiles of each individual ARG screening tool

**Figure 2:**
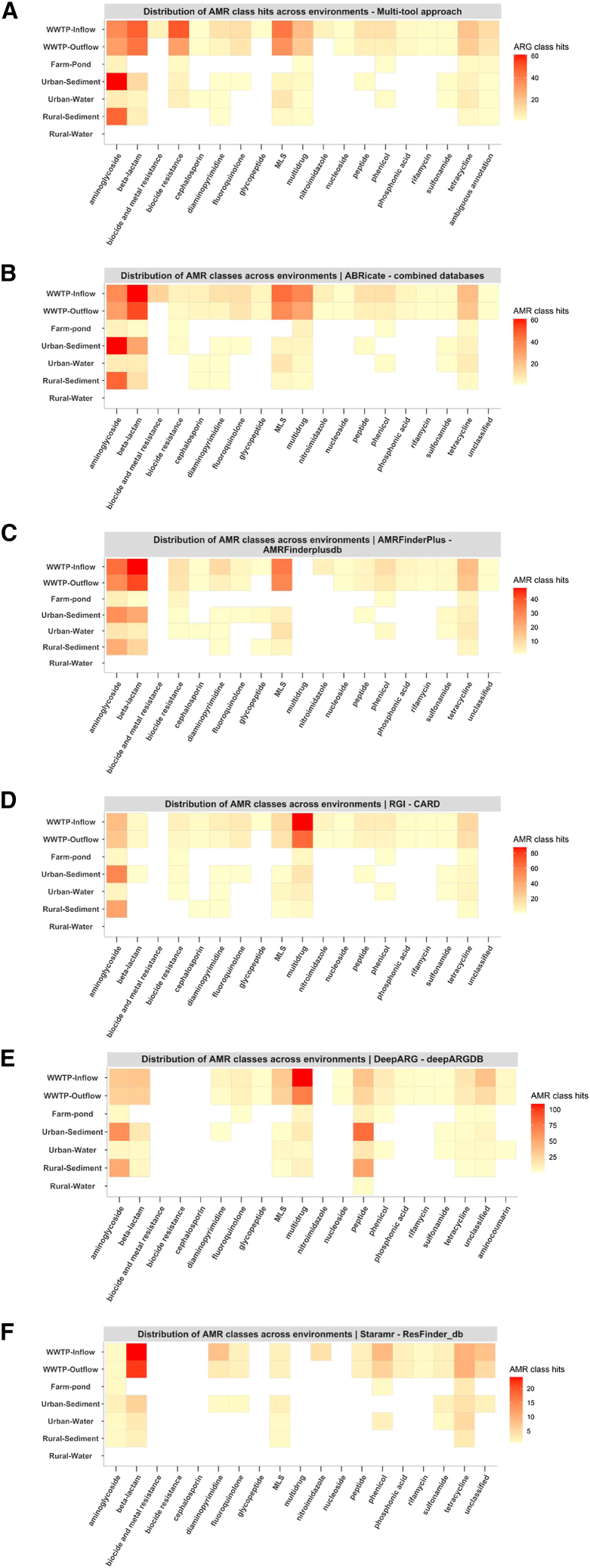
Distribution of ARGs hits predicted in each environment and classified by the antibiotic resistance class for each of the ARG screening tools included in the study Panels indicate different screening tools (A) multi-tool approach, (B) ABRicate, (C) AMRFinderPlus, (D) RGI, (E) DeepARG, and (F) Staramr. ORFs were annotated for ARGs separately by each ARG screening tool and assigned to a certain AMR class. Each square in the heatmap corresponds to the total number of ARG sequences linked to an AMR class and environment (sample group). The drug class biocide resistance and biocide and metal resistance are part of MEGAres database but is not included in the CARD database.

Results of the comparison between the detection profile of the multi-tool approach and the detection profiles resulting from using each tool individually are shown in Figures 2A–2F. The maximum number of ARG hits detected per environment and AMR class differed between ARG screening tools: with DeepARG (based on machine learning) the highest number of 972 ARG hits was detected, followed by ABRicate with 749 ARG hits, RGI with 578 ARG hits, AMRFinderPlus with 479 ARG hits, and finally Staramr with 264 ARG hits. The multi-tool approach reported 730 ARG hits per AMR class and environment, often detected by different combinations of tools and databases. There were 11 ARG classes out of 18, i.e., aminoglycosides, beta-lactams, diaminopyrimidine, fluoroquinolones, MLS, peptide, phenicol, phosphonic acid, rifamycin, sulfonamide, and tetracycline, which were reported by all ARG tools used in this study.

The tools that performed most similarly to the multi-tool approach (Figure 2A; Table 1) by detecting a higher number of shared ARG classes were ABRicate, followed by RGI (which shared 17 ARG classes), AMRFinderPlus (which shared 16 ARG classes), DeepARG (which shared 15 ARG classes), and Staramr (which shared 12 ARG classes). Both ABRicate and AMRFinderPlus detected a higher prevalence of aminoglycosides, beta-lactams and MLS in the WWTP in- and outflow and a higher number of aminoglycosides in the urban and rural sediments.

**Table 1:** Detection profile of the 5 ARG screening tools used in the multi-tool approach

ARG screening tool	Total drug classes detected	No. of drug classes undetected	Undetected drug classes compared to multi-tool approach	Total no. of ARG hits	Distinct drug classes detected
**ABRICATE**	18	0	–	749	–
**RGI**	17	1	biocide and metal resistance	578	–
**AMRFinderPlus**	16	2	biocide and metal resistance and multidrug classes	479	–
**DeepARG**	15	4	biocide and metal resistance, biocide resistance, cephalosporin and nitroimidazole	972	aminocoumarin
**Staramr**	12	6	biocide and metal resistance, biocide resistance, cephalosporin, glycopeptide, multidrug and nucleoside	264	–
**Multi-tool approach**	18	1	aminocoumarin	730	biocide and metal resistance

The following fields are listed for each tool: total number of ARG classes detected, number of detected classes (compared to the 18 ARG classes detected by the multi-tool approach or compared to all the single tool annotation in the case of the last row corresponding the multi-tool approach), undetected ARG classes when compared to the multi-tool approach, total number of ARG hits and distinct ARG classes detected (compared to the multi-tool approach in all cases except the last row, which refers to the multi-tool approach).

From all five tools, only DeepARG detected a unique drug resistance class, the aminocoumarin class (in the WWTP in- and outflow and urban water), which was not reported by the multi-tool approach or any other tools.

### Differences in mean ARG counts across different environments

**Figure 3:**
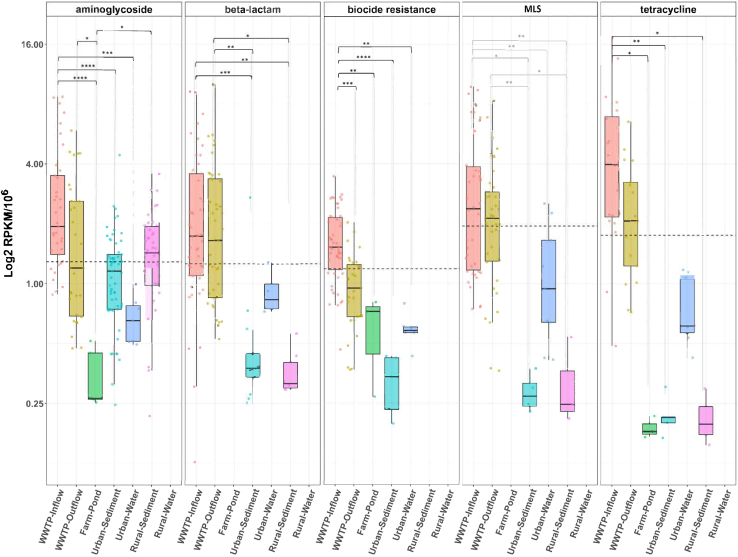
AMR class abundance comparison for those AMR classes that showed significant differences between environments ORFs were annotated for ARGs separately with the multi-tool approach and assigned to a certain AMR class. The mean rank of the RPKM-SCMG counts was computed by mapping the high-quality reads to the ORFs. A logarithmic scale has been used to represent the RPKM-SCMG counts in the Y axis. The dashed line represents the average of the mean rank of the RPKM-SCMGs counts per each AMR class. *p*-values significance {∗ < 0.05, ∗∗ < 0.01, ∗∗∗ < 0.001, ∗∗∗∗ < 0.0001} computed with Dunn test for pairwise multiple- comparison.

Four ARG classes (aminoglycosides, beta-lactams, MLS, and tetracycline) and the drug class of biocide resistance showed differences in the mean rank of the RPKM-SCMG counts between different environments, according to the Kruskal-Wallis test, the Dunn’s test for pairwise multiple comparisons and computing Bonferroni adjusted *p* values (Figure 3).

For the aminoglycoside AMR class, the mean rank of ARG counts in the WWTP inflow was significantly higher than in all other environments, except for WWTP outflow and rural sediments (adjusted *p* value <0.001, Tables S4 and S5). The mean rank of aminoglycoside ARG counts was significantly lower in the farm pond when compared to the WWTP inflow (adjusted *p* value <0.05, Tables S4 and S5). In the case of beta-lactams, the mean ranks of both WWTP in- and outflow counts were significantly higher than in urban (adjusted *p* value <0.0001, Tables S4 and S5) and rural sediments (adjusted *p* value <0.01, Tables S4 and S5). For MLS, the mean rank of WWTP inflow was significantly higher than urban water (adjusted *p* value <0.05, Tables S4 and S5). Another drug class that showed statistical differences among environments were genes conferring resistance to biocides, which had a higher mean rank count in WWTP inflow compared to urban water (adjusted *p* value <0.001, Tables S4 and S5), WWTP outflow (adjusted *p* value <0.01, Tables S4 and S5) and urban sediments (adjusted *p* value <0.01, Tables S4 and S5). Finally, for tetracyclines, the ARG mean rank count in the WWTP inflow was significantly higher than in urban sediments (adjusted *p* value <0.01, Tables S4 and S5), farm pond (adjusted *p* value <0.05, Tables S4 and S5), and rural sediments (adjusted *p* value <0.05, Tables S4 and S5). No ARGs were detected in rural water for any of the ARG classes predicted using the multi-tool approach.

**Figure 4:**
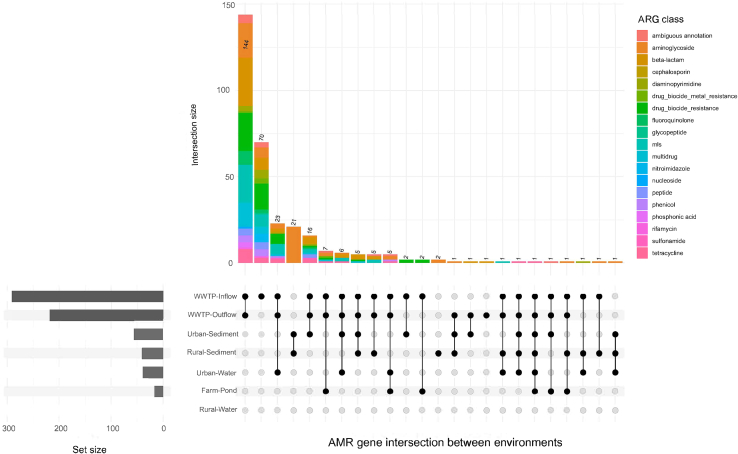
Number of ARGs intersected between the different environments ORFs which were annotated for ARGs were linked to the different environments by mapping the high-quality reads of each sample back to the ORFs. Each bar in the top panel corresponds to a set of ARGs that are unique to a particular combination of environments (indicated by dot and lines). The number ARGs per each environment is depicted at the bottom left of the plot by horizontal bars.

While the statistical tests show which ARG classes differ in abundance across environments, they do not indicate whether the same specific ARGs are shared between compartments. Therefore, we decided to examine the ARG overlap across urban compartments. The amount of ARGs shared was much higher in WWTP in- and outflow than in lakes (Figures 4 and S3). In total, 144 ARGs were common between the WWTP in- and outflow, and 70 ARGs were found exclusively in the WWTP inflow. This demonstrates that from a total of 214 ARG hits detected exclusively in the WWTP in- and outflow, 32.7% of the ARGs in the inflow were filtered out during the WWTP treatment (Figure 4; Table S3). Twenty-three ARG hits were only present in the WWTP in- and outflow and urban water. Twenty-one ARG hits, mainly associated to aminoglycoside resistance, were found simultaneously between urban and rural sediments. Sixteen ARG hits were shared between urban sediments and WWTP in- and outflow. ARGs shared between freshwater environments and WWTP in- and outflow mostly belonged to the drug classes aminoglycosides, beta-lactams, biocide resistance, MLS, and tetracyclines. Fewer ARG hits that were identified simultaneously in multiple environments were shared between WWTP in- and outflow and other freshwater environments or the farm pond (Table S6).

**Figure 5:**
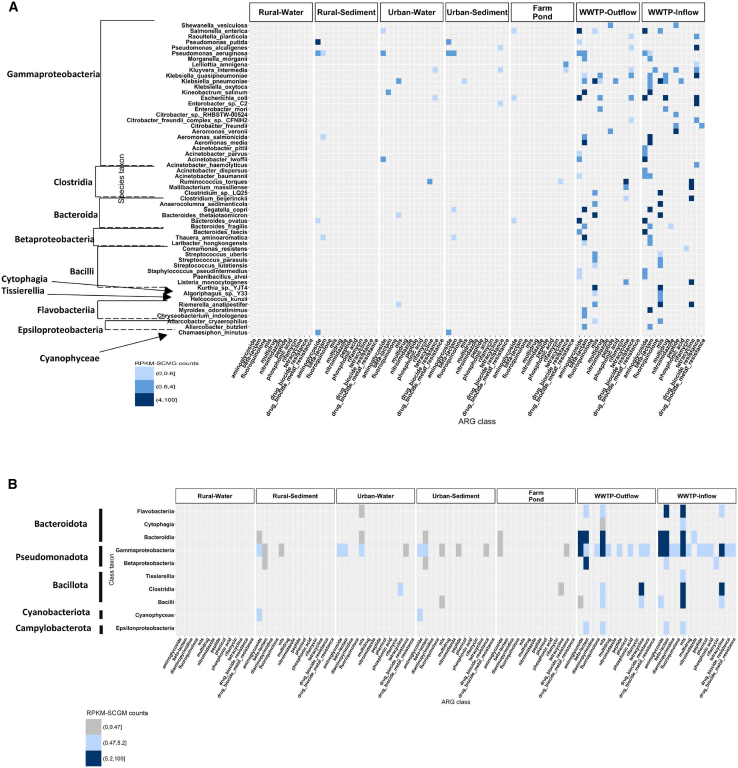
Association between the taxonomic rank and the AMR class annotation in each of the environments (A and B) corresponds to different taxonomic ranks: (A) species and (B) class level. ORFs were annotated for ARGs and for taxonomic annotation using Kraken2 with the core_nt Database. The mean rank of RPKM-SCMG counts was computed by mapping the high-quality reads to the ORFs. Each square in the heatmap corresponds to the ARG mean rank of the RPKM-SCMG associated to a specific taxon and a specific environment.

To relate the overlaps in ARG profiles between environments to the microbial community composition, we next assessed the taxonomic profiles across samples (Figure 5A). Gammaproteobacteria constituted the most predominant taxa detected in all environments, except in rural water, where no taxon was annotated (Figure 5A). WWTP in- and outflow exhibited higher RPKM-SCMG counts than samples from any of the freshwater environments. Up to 6 different phyla were annotated in the WWTP in- and outflow but no annotation was found for cyanobacteria, which in our study was detected in both urban and rural sediments. Gammaproteobacteria were mainly associated with aminoglycosides, beta-lactams, MLS, and tetracycline in both WWTP in- and outflow. Biocide resistance genes were also predicted from WWTP in- and outflow at levels higher than 4 RPKM-SCMG counts (1st quartile: 0.495 RPKM-SCMG, median: 1.67 RPKM-SCMG, and 3rd quartile: 5 RPKM-SCMG). Up to four different phyla could be detected in the sampled freshwater systems (i.e., bacteroidota, pseudomonadota, bacillota, and cyanobacteriota). Cyanobacteriota could be annotated from rural and urban sediments and urban water, showing a profile similar to that of the farm pond, with *Clostridia* associated with tetracycline. Five taxonomic classes were detected in urban sediments, four taxonomic classes in urban water and rural sediments, three in the farm pond and no taxonomic classes from any rural surface water samples.

At the species level, WWTP in- and outflow had a higher diversity of species and ARG classes than any other sample type (Figure 5B). Several species were found to be enriched in both WWTP in- and outflow, e.g., *Pseudomonas aeruginosa*, which was also reported in rural sediments and urban waters and sediments (associated with aminoglycoside and beta-lactams ARGs), *Escherichia coli* (*E. coli*) (associated with aminoglycoside ARGs), *Klebsiella pneumoniae* (associated with MLS ARGs), *Ruminococcus torques* (associated with tetracycline ARGs), *Salmonella enterica* (associated with beta-lactam ARGs). For some of the taxa mentioned previously, we could detect up to four ARGs, which were annotated to a particular taxon and environment, potentially indicating the presence of multi-resistant bacteria. In the case of *Pseudomonas aeruginosa*, both aminoglycoside and beta-lactam ARGs were annotated in rural sediments, urban sediments, and the WWTP in- and outflow. Taxonomic annotation indicated that among all sampled freshwater systems, urban water accounted for the highest diversity of species (ten different taxa) linked to four classes (Gammaproteobacteria, Clostridia, Bacteroidia, and Flavobacteria) and predominantly associated with aminoglycoside and MLS resistance. In the farm pond, rural and urban sediment samples also showed aminoglycoside as a prevalent AMR class, associated with different classes. In both urban and rural sediments, different species, such as *Pseudomonas aeruginosa*, *Aeromonas salmonicida*, and *Thauera aminoaromatica*, were associated with the beta-lactam AMR class.

**Figure 6:**
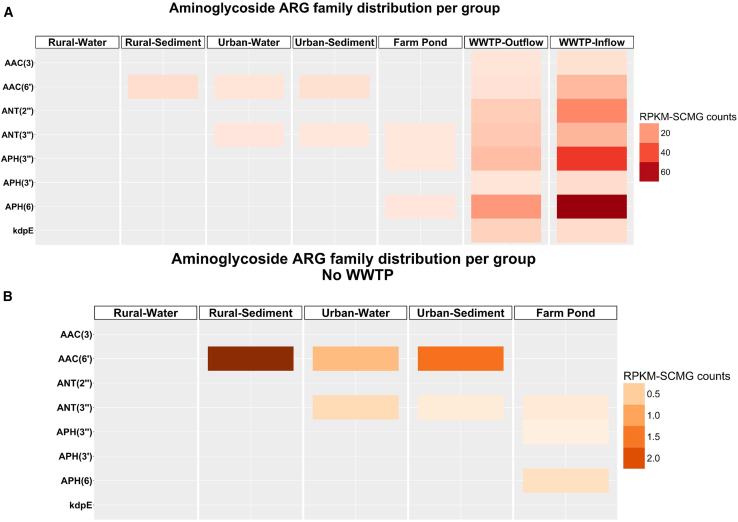
Distribution of aminoglycoside resistance gene families across the different environments (A and B) correspond to different environments: (A) including the wastewater treatment plant (WWTP) or (B) excluding the WWTP. Open reading frames (ORFs) were annotated for antibiotic resistance genes (ARGs), filtered by aminoglycoside class and grouped in aminoglycoside gene families according to CARD database (Alcock et al., 2023). The mean rank of the RPKM-SCMG counts was computed by mapping the high-quality reads to the ORFs. Each square in the heatmap corresponds to mean rank RPKM-SCMG counts associated to an aminoglycoside gene family and environment.

The two most abundant aminoglycoside families in WWTP in- and outflow were APH(3″) and APH(6), both more abundant in the WWTP in-than outflow (Figure 6A). The farm pond shows a different pattern of aminoglycoside family distribution when compared to all other freshwater environments, sharing the APH(3′) and APH(6) families with WWTP in- and outflow. The abundance of the aminoglycoside families in the sampled freshwater systems (Figure 6B) highlights that rural sediments harbor the highest mean RPKM-SCMG counts for AAC(6′), which is the most abundant aminoglycoside family predicted from all sampled freshwater environments. AAC(6′) in urban sediment showed the second highest mean abundance, which was higher when compared to any other mean count from the aminoglycoside gene families predicted from urban water or the farm pond.

**Figure 7:**
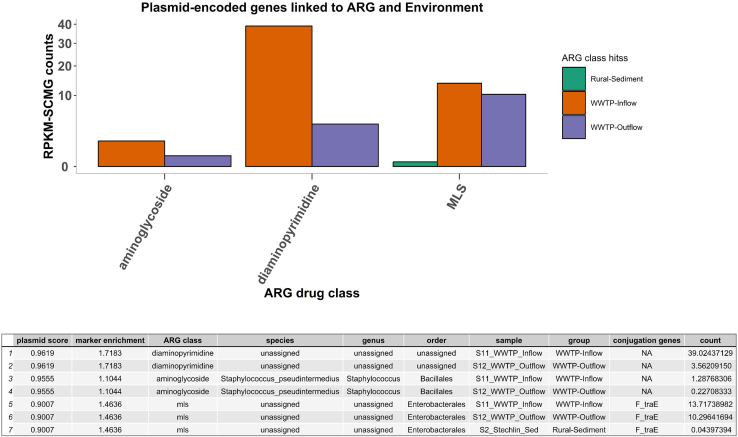
Number and sample group distribution of Open reading frames (ORFs) annotated simultaneously as ARGs and Mobile genetic elements (MGEs) Open reading frames (ORFs) were annotated for antibiotic resistance genes (ARGs) and for mobile genetic elements with geNomad v1.8.1. Only those AMR classes and environments for which at least one hit was present are represented in the figure. The ∗ indicates that F_traE conjugation genes were detected in the plasmid-encoded genes.

Annotations of open reading frames (ORFs) containing plasmid sequences were conducted using geNomad, which identifies and classifies plasmids from sequencing data by mapping proteins to reference genomes of both isolates and uncultivated species. A total of three plasmid sequences could be predicted in three ORFs associated with WWTP in- and outflow or rural sediments (Figure 7). Out of all the ORFs linked to plasmids, one ORF was annotated as diaminopyrimidine ARG (detected in WWTP inflow and outflow), another ORF as an aminoglycoside ARG (detected in WWTP in- and outflow) and the last ORF as MLS ARG (detected in WWTP in- and outflow, and “rural” Lake Stechlinsee sediments). The aminoglycoside ARG linked to plasmid sequences detected in the WWTP in- and outflow may have been hosted by *Staphylococcus pseudointermedius,* as predicted by geNomad.

WWTP in- and outflow showed the highest ARG abundance (RPKM-SCMG) for ARGs linked to plasmid sequences (Figure 7), including diaminopyrimidines, MLS, and aminoglycosides. Furthermore, only the ORF sequence annotated as MLS ARG appears to code for conjugation genes (F_traE). No plasmid sequences were detected in ORFs from urban and rural water or the farm pond. No viral sequences were detected in any ORFs containing ARGs in any of the samples.

## Discussion

### Comparison between single-tool and multi-tool ARG annotation

Correct assessment of an ARG tool’s performance relies on determining its prediction accuracy, with low rates of false negatives and false positives. Several studies compared the accuracy of multiple ARG tools using phenotypic testing or ARGs reported in the literature^22^^,^^27^^,^^31^^,^^32^ Among these, some reported minor differences between the tools used, stating that minimal differences were observed when analyzing assembled or raw reads. If the AMR prediction profiles in each study are summarized (Table S2), one can observe that differences in the types of input samples between studies significantly impact the number of ARG classes predicted by the same tool; for example, the number of ARG classes detected with DeepARG in this study was twice that in Marini.^27^ Other differences can be observed when comparing the performance of one tool among different studies; Gomes^32^ report that ABRicate predicts the highest AMR class diversity, whereas Wissel^22^ show that ABRicate prediction capacity is outperformed by many other tools, and Marini^27^ report a higher accuracy of ARG classes by K-mer-based antibiotic resistance gene analyzer (KARGA). However, there is no reference for ARG detection by independent methods, and the accuracy of each tool relative to the others has not been comprehensively assessed.

To this end, our multi-tool approach successfully addressed annotation discrepancies across tools and databases, thereby increasing the robustness of ARG prediction. Except for ABRicate, none of the individual tools in this analysis detected as many ARG classes (18) as the multi-tool approach. Previous studies using other multiple ARG tools and databases have also reported a broader detection of environmental ARGs than a single-tool approach.^30^^,^^32^ Single-tool methods are biased toward clinical ARGs^24^^,^^25^ and, while different ARG databases can share multiple ARGs and cover similar ARG classes, others, such as the CARD or ResfinderFG v.2.0 databases, hold unique sets of ARGs and ARG classes, including quinolones, sulfonamides, and trimethoprim. The broad ARG diversity predicted by ABRicate suggests that using multiple, diverse databases rather than multiple algorithms could significantly increase the coverage of AMR diversity detected in the environment. In this regard, using ABRicate with all ARG databases (NCBI, AMRFinderPlus, CARD, Resfinder, antibiotic resistance gene-annotation (ARG-ANNOT), and microbial ecology group antimicrobial resistance (MEGARES)) updated to its latest version appears to be the best tool for screening environmental ARGs.

### Confidence thresholds and detection bias

To ensure a minimum confidence level for ARG prediction, we required that at least two tools share the same AMR class annotation. One potential drawback of the approach is the potential to underestimate the diversity of predicted ARG classes. In our study, one AMR class (aminocoumarin) was excluded from the analysis because the corresponding ARGs were reported by only one tool, DeepARG. However, these annotations may not be false positives and could provide relevant information about less well characterized ARGs detectable by only a few specific tools. Such cases should be further investigated in the lab with PCR-based techniques and phenotypic analyses, and their addition reconsidered, on a case by case basis.

It is also important to highlight other limitations to consider when comparing ARG profiles. In our analysis, different sequencing depths were achieved between water samples (rural and urban waters and farm pond) and sediment and WWTP samples, which could introduce bias into estimates of the ARG diversity difference. Nevertheless, the high abundance and diversity of ARGs observed in urban water, and the comparisons between the WWTP and the sediment samples were robust enough to counter those potential effects.

Despite recognizing the obvious difference in sequencing depth, the rarefaction curves in the observational domains indicate a lower detection of ARGs in water samples than in sediments when using the unfiltered ARG dataset (Figure S1A), but instead a very similar or even slightly higher detection rate in filtered datasets (Figure S1B). The same can be inferred for ORFs and COGs (Figure S2), where, again, for COGs, the number of ARG sequences in water samples is higher than in sediments. The number of sequences in water samples (*ca.* 100), albeit lower than in other environments, appears to be adequate to indicate the observed potential trend in values as a function of sequencing depth, therefore minimizing the relative importance this potential bias might introduce.

### Differences and similarities in ARG composition between environments

Previous studies have shown higher concentrations of ARGs in surface water compared to lake sediments.^4^^,^^33^ We also observed the same pattern across four of five drug classes (beta-lactams, MLS, tetracyclines, and biocides). In the case of aminoglycosides, the higher abundance in urban and rural sediments compared to urban water may reflect the fact that sediments act as archives and long-term storage for ARGs that were present in the water.^34^ During the 1950s and 1960s, European states approved national regulations on antibiotic use in animals, which may have led to environmental contamination of water with aminoglycoside antibiotics.^35^ In addition, soil microbiotas are known to be important producers of aminoglycoside antibiotics, which could result in higher aminoglycoside resistance in sediment.^36^ Antibiotic signatures for lake water and sediments have been characterized for multiple lakes in China, showing that in water bodies, sulfonamides and quinolones are the most prevalent ARG classes, whereas in sediments, tetracyclines and quinolones are predominant.^37^^,^^38^ ARG contamination sources linked to animal husbandry or agricultural practices are confirmed in our study by a higher abundance of tetracycline resistance in urban surface water. We detected abundant sulfonamide resistance genes in the WWTP in- and outflow as well as in water and sediment samples from urban lakes, which agrees with former studies attributing the high prevalence of sulfonamide resistance to wastewater and MGEs in lakes with high anthropic impact, e.g., clinical and veterinary.^38^^,^^39^^,^^40^ Instead, the lack of sulfonamide ARGs in rural lakes or the farm pond supports the association of this AMR resistance with the presence and interaction with human populations.

Our results also showcase how specific activities in urban environments (e.g., Lake Müggelsee surface waters reporting five to ten times more classes than the other two urban lakes) might account for a large, if not the most important, share of the differences in ARG classes present namely fishing, shipping, recreation, and as a source of drinking water.^41^ Conversely, limited urbanization in the proximity of lakes might account for similar AMR class diversity (i.e., urban lakes Haussee and rural lakes Dagowsee and Stechlinsee). In particular, diaminopyrimidine resistance detected in the sediments from Lake Dagowsee could be linked to the previously reported use of trimethoprim for duck and carp raising and for sewage disposal during the period 1949–1990.^42^ And both lakes Dagowsee and Stechlinsee, and their immediate vicinity, were used during the period 1949–1990 for bird/fish farming and mass tourism, respectively.^42^^,^^43^

Our study except for aminoglycoside consistently supports that aquatic urban sediments harbor a higher ARG abundance when compared to rural aquatic environments,^38^^,^^44^^,^^45^ and that urban water bodies exhibit a higher ARG diversity and abundance when compared to rural waters.^38^^,^^40^^,^^46^^,^^47^

However, few studies, such as this one, have simultaneously compared urban and rural water.^6^^,^^8^^,^^48^^,^^49^

Aminoglycosides, tetracycline, and phenicol ARGs were also detected in the farm pond, consistent with previous studies that reported the same ARG classes in water samples collected from pig farms.^50^ Aminoglycosides, phenicols, and tetracyclines, which are all widely used in animal husbandry, were also found in the farm pond sample.

In summary, ARG abundances were significantly different across environments (Figures 1A and 1B), with, for example, aminocoumarins in urban sediments having very low relative abundances compared to most environments (Figure 1B).

### Bacterial community shifts and ARG patterns in lakes and WWTPs

In contrast to the ARG diversity observed in our study, the majority of the shared bacterial diversity was highest between urban and rural lakes, indicating a decoupling from ARG diversity. One possible explanation could be that most ARG-carrying bacteria are concentrated in water sources derived from hospital effluent and human wastewater, as in most cases, bacteria were exposed to antibiotics. The broader diversity of conditions found in urban freshwater lakes (including substrates, temperatures, and other biotic and abiotic factors) could lead to greater diversity of bacterial taxa and functional capabilities. This could explain the slightly greater taxonomic diversity found in bacterial communities from urban lakes when compared to rural ones. Rarefaction curves, however, suggested that a higher sequencing depth would be required to fully characterize surface water ARG diversity, as overall DNA recovery was lower than in sediments.

As expected, the WWTP comprised a much larger AMR diversity followed by urban samples than other environments, with almost double the number of ARG classes than the environment. Sixteen of the 18 ARG classes detected in the WWTP inflow were also found in the WWTP outflow, and no ARG class showed significant differences in abundance between these locations. These results indicate that the wastewater treatment does not fully remove ARG-containing bacteria despite yielding a large bacterial concentration reduction and more than 50% of the ARG hits (i.e., 76 out of 144) were not detected in the WWTP outflow.

Glycopeptide- and nitroimidazole-resistance ARGs were found in the WWTP in- but not in the outflow. A possible explanation is that bacteria carrying these ARGs in the WWTP inflow were removed or diluted after the WWTP treatment. Although some specific ARG classes are reported to be largely removed during wastewater treatment,^51^ WWTPs cannot differentiate among or selectively filter out specific ARGs, and detection may be related to the initial abundance of a given AMR class. Despite being identified in this study, the low prevalence of these antibiotics in the environment could have led to low antibiotic resistance detection in the WWTP inflow, which, in turn, might have resulted in no detection in the WWTP outflow.

The higher number of unique ARGs in the WWTP inflow may be explained by wastewater treatment affecting the bacterial community in the outflow.^3^ The removal of bacterial cells, taxa, and associated ARGs during the treatment process may lead to ARG levels below the detection limit, even with deep sequencing. Although the abundance and diversity of ARGs did not differ significantly between WWTP in- and outflow, we observed a reduction in overall ARG abundance after WWTP treatment, consistent with the previous studies.^52^^,^^53^

The overall distribution of bacterial taxa across the different samples is consistent with our previous study using non-pooled samples.^3^ In the present study, we pooled three samples per site and performed metagenomics shotgun sequencing using Illumina short reads. This pooling of samples may explain some of the discrepancies between the previous and current analyses, particularly for bacterial taxa at low abundance, which are easier to detect by PCR-based approaches.

### Plasmid-linked ARGs and ORF-level host associations

Multiple associations between ARG classes and bacterial taxa could be established at the ORF level. Among all drug classes, aminoglycosides, MLS, beta-lactams, or tetracyclines predominated and were associated with different bacterial taxa. Most ARG classes were enriched in WWTP in- and outflow but were also found in urban and rural sediments and the farm pond. We also observed that these drug classes are linked to specific pathogens, as reported in other studies.^54^^,^^55^ For example, *Klebsiella pneumoniae* (beta-lactams, MLS, multidrug, and phosphonic acid ARGs) was detected exclusively in the WWTP in- and outflow. *Pseudomonas aeruginosa* in the rural sediments was associated with aminoglycosides and beta-lactam.

Three different ORFs potentially linked to plasmid sequences could also be associated with three ARGs, i.e., aminoglycosides, MLS, and diaminopyrimidine in WWTP in- and outflow, with the MLS plasmid also found in rural sediment, which was the only case where the conjugation genes F_traE were observed. Both aminoglycosides and MLS ARGs have been previously associated with plasmids sequenced from heavily contaminated riverine waters and sediments in Costa Rica.^47^ F_traE genes are thought to be involved in the resolution of plasmid DNA replication intermediates.^56^^,^^57^ In this study, we did not screen for integrons and transposon sequences as our main research question was not aimed at evaluating the direct transmission of ARGs between environments. However, these MGEs are of great importance in the transmission of ARGs and we strongly recommend screening for such sequences in any follow-up, or other study screening ARGs from metagenomic samples. Two aminoglycoside ARG gene families could be detected in both urban water and sediments, namely *ANT(3″)* and *AAC(6′)*, the latter also found in rural sediments. *ANT(3″)* is the most common class of *ANTs* enzymes found, ubiquitous across different biomes, including WWTP, freshwater aquatic systems, pig farms, as well as human samples.^58^^,^^59^^,^^60^^,^^61^^,^^62^ Two aminoglycoside resistance genes, i.e., *AAC(6′)-IIa* and *AAC(6′)-32*, were also found in rural and urban lake sediments, and *AAC(6′)-II* was found in rural and urban sediments as well as in urban water (Figure S4). Other aminoglycoside ARG genes such as *AAC(6′)-I*, *AAC(6′)-Ib*, and *AAC(6′)-IE* could not be detected in the rural sediments.

The widespread use of aminoglycoside antibiotics for the clinical treatment of human infections is associated with increased abundance of these ARGs in municipal wastewater. Aminoglycoside antibiotics are commonly used to prevent bacterial diseases in animal husbandry, apiculture, and aquaculture.^63^^,^^64^ The fact that most aminoglycoside ARGs in urban and rural lakes are also reported in wastewater indicates that aminoglycoside antibiotics application in clinical settings and/or food production tends to enrich a similar pattern of aminoglycoside ARGs in the aquatic environment.^65^

WWTPs collect and concentrate ARGs from multiple urban water sources, whereas the farm pond was not connected to any WWTP. Aminoglycoside antibiotics, such as gentamicin and tobramycin, are commonly used in German neonatal intensive care units (NICUs) to treat Gram-negative infections. At the same time, in 2015, the sales of aminoglycosides, when aggregating 30 European countries, made up for 3.5% of the total sales of antimicrobials for food production, and the sixth most used antimicrobial class for veterinary medicine.^63^^,^^65^^,^^66^ This may explain the relatively high overlap of aminoglycoside ARGs between the WWTPs and the farm pond (three gene families shared) compared to WWTP vs. lake water and sediments (two gene families shared).

### Multi-tool ARG screening for environmental resistome annotation

The current study highlights the importance of using a multiple ARG screening approach to detect ARGs in aquatic environments. Our study demonstrated that using ABRicate with all ARG databases (NCBI, AMRFinderPlus, CARD, Resfinder, ARG-ANNOT, and MEGARES) updated to their latest version would potentially be the best tool for screening environmental ARGs. Exceptionally, the use of additional single tools might be advised if a specific class not covered were needed. WWTPs clearly have the highest levels of ARG classes compared to urban and rural environments and could at times act as a potential source of AMR inflow. The results of the current study support the central role of sediments, particularly those in the more contaminated settings (here, WWTPs), as an important reservoir for ARGs and showcase the high number of ARG classes detected in both urban and rural lake sediments. The current study, to the best of our knowledge is the first to report an ORF sequence linked to F_traE conjugation genes associated with MLS in a natural aquatic habitat. That conjugation genes could be detected in this ORF and not in others suggests a larger distribution of this plasmid (not only in WWTP in- and outflow water but also in rural sediments) when compared to the occurrence of the other two detected ORFs linked to plasmid sequences (exclusively associated with WWTP in- and outflow).

Although validation using methods such as PCR-based assays remains essential, future enhancements of databases that better capture environmental AMR diversity will improve the precision and accuracy of deep sequencing-based approaches, ultimately strengthening environmental AMR surveillance.

### Limitations of the study

As stated in the “discussion” section, different sequencing depths were obtained between water samples (rural and urban waters and farm pond) and sediment and WWTP samples, which could have introduced a bias into estimates of the ARG diversity difference. Nevertheless, the high abundance and diversity of ARGs observed in urban water, and the comparisons between the WWTP and the sediment samples were robust enough to counter those potential effects. The assessment of the impact of the differences (Figure S1) showed a high ARG coverage for sediment and WWTP samples but a potential relative underestimation of ARGs from the water samples.

## Resource availability

### Lead contact

Further information and requests should be directed to and will be fulfilled by the lead contact, Prof. Alex D. Greenwood (greenwood@izw-berlin.de).

### Materials availability

This study generated no new materials.

### Data and code availability

• This study did not generate any new unique code.
• Raw whole-genome sequencing (WGS) data were deposited into the European Nucleotide Archive (ENA), under the accession number PRJEB98077.
• Any additional information regarding the data reported in this publication will be made available by the corresponding author upon request.

## Acknowledgments

We thank Danny Ionescu, Jason Woodhouse, Guilherme Neumann, Dorina Meneghini, and Rafael Cuadrat for their computational assistance in this study. We thank Karin Hönig and Solvig Pinnow for technical support. We thank Jaffer Dar, Sabreen Samuel Ibrahim Dawoud, and Richard Mugani for their support in the sampling and other technical help. PDY, ADG, and HPG were supported by a project grant (IRG 3-Water) from the Leibniz Research Alliance “INFECTIONS’21 - INFECTIONS in an Urbanizing World - Humans, Animals, Environments” funded by the 10.13039/501100001664Leibniz Association, Germany (SAS-2015-FZB LFV). We thank two anonymous reviewers for the useful comments and edits on an earlier version of this study.

## Author contributions

P.D.Y., sampling, methodology, data curation, formal analysis, visualization, writing original, and writing – review and editing; L.Z., data curation, conceptualization, methodology, formal analysis, and writing – review and editing; J.A.G., methodology, data curation, and writing – review and editing; D.N., sampling, methodology, and data curation; N.A., conceptualization and writing – review and editing; H.-P.G., funding acquisition, conceptualization, methodology, supervision, and writing – review and editing; A.D.G., funding acquisition, conceptualization, methodology, supervision, and writing – review and editing.

## Declaration of interests

The authors declare that they have no known competing financial interests or personal relationships that could have appeared to influence the work reported in this paper.

## STAR★Methods

### Key resources table

**Table:** 

REAGENT or RESOURCE	SOURCE	IDENTIFIER
**DNA extraction kit**		

NucleoSpin® Soil kit	Macherey	740472.50

**Deposited data**		

Metagenomic data (raw reads)	ENA	PRJEB98077

**Software and algorithms**		

Abricate version 1.0.1	–	https://github.com/tseemann/ABRICATE
Amrfinder version 3.11.4	https://doi.org/10.1038/s41598-021-91456-0	https://github.com/ncbi/amr
DeepARG version 1.0.2	https://doi.org/10.1186/s40168-018-0401-z	https://github.com/gaarangoa/deeparg
RGI version 5.2.1	https://doi.org/10.1093/nar/gkac920	https://github.com/arpcard/rgi
Staramr version 0.9.1	https://doi.org/10.3390/microorganisms10020292	https://github.com/phac-nml/staramr
NCBI AMRFinderPlus database (db)	https://doi.org/10.1128/AAC.00483-19	–
CARD db	https://doi.org/10.1093/nar/gkw1004	–
Resfinder db	https://doi.org/10.1093/jac/dks261	–
ARG-ANNOT	https://doi.org/10.1128/AAC.01310-13	–
PlasmidFinder	https://doi.org/10.1128/AAC.02412-14	–
MEGARES 2.00	https://doi.org/10.1093/nar/gkz1010	–
Deeparg db	https://github.com/gaarangoa/deeparg	1.0
Amrfinder db	https://github.com/ncbi/amr	2023-02-23.1
RGI db	https://github.com/arpcard/rgi	3.2.9
Staramr db	https://bitbucket.org/genomicepidemiology/resfinder_db/src/master/	2022-05-24

**Other**		

Illumina sequencing	Illumina	Illumina PE150 HiSeq X

### Method details

#### Study sites and sample collection

Lakes Müggelsee and Weisser See in Berlin (the capital of Germany with an area of 891.1 km2 and 3.7 Mio inhabitants) and Lake Haussee (located in the small city of Feldberg in Mecklenburg-Vorpommern, in the state of Mecklenburg-Vorpommern) were exposed to pronounced anthropogenic impacts due to previous wastewater input,^67^ making them comparable to lakes in bigger cities and are thus defined as urban lakes. None of the urban lakes received direct wastewater input during the sampling period. The two rural lakes, Lake Dagowsee and Lake Stechlinsee are located in a forested nature reserve in Northern Brandenburg and have little anthropogenic impact, surrounded by only 383 inhabitants in the villages of Dagowsee and Neuglobsow. All lakes originate from the last ice age but greatly vary in their present environmental status.

Untreated raw inflow water and treated outflow were sampled from a municipal wastewater treatment plant (WWTP) processing the waste of *ca.* 3.5 million citizens of Berlin (Germany) in 2016.^68^ This WWTP processes a negligible amount of industrial wastewater. Samples were collected at the in- and outflow of the WWTP. A water sample from a farm pond was collected in the municipality of Groβ Kreutz, which is located in the district Potsdam-Mittelmark (Brandenburg, Germany) and has a population of 8,948 inhabitants. The farm pond is located 6 km away from a wastewater treatment plant and more than 1 km distance from Groβ Kreutz itself. The characteristics of all five lakes, the wastewater treatment plant, and the farm pond are shown in Table S1.

Wastewater in- and outflow, lake surface water, and sediment samples were collected every three months in 2016 from two locations in lake Weisser See and three different locations in lakes Müggelsee, Haussee, Dagowsee and Stechlinsee. Another set of water samples was collected from a farm pond (Groβ Kreutz). Further details on sample collection are described in Numberger.^3^

Samples were grouped as follows:

1. Rural-Water and Rural-Sediments (water or sediments from lakes Dagowsee and Stechlinsee), 2. Urban-Water and Urban-Sediments (water or sediments from lakes Haussee, Müggelsee and lake Weisser See), 3. Water from a farm pond in Groß Kreutz and 4. WWTP (in- or outflow).

#### DNA extraction and metagenomic sequencing

DNA from the water was extracted from Sterivex filters (EMD Millipore, Darmstadt, Germany) using the QIAamp DNA mini kit (Qiagen, Hilden, Germany) following the protocol for tissues with some modifications. WWTP and sediment samples showed both a similar semi-solid nature, hence, the DNA from both environments was extracted using the NucleoSpin Soil kit ref. 740472.50 (Macherey 180 Nagel, Düren, Germany), which is suitable for sediment, solid and sludge samples. More details on sample collection are given in Numberger.^3^ Replicates from each site (considering WWTP in- and outflow as separate sites) were pooled to reduce the impact of spatial and temporal heterogeneity. Subsequently, the DNA was sequenced using Illumina PE150 HiSeq X, which resulted in sequencing depths per sample ranging from 5 to 390 million reads. The assessment of the impact of the differences (Figure S1) showed a high ARG coverage for sediment and WWTP samples but a potential relative underestimation of ARGs from the water samples.

#### Bioinformatic analyses

The paired reads sequences from the Illumina platform were trimmed, filtered and PhiX and human reads were removed using BBtools v38.96^69^ following the hyperparameter recommendations in Methods in Microbiomics.^70^ The resulting high-quality retained reads had a quality score >20. Reads were assembled into contigs using Megahit *v1.2.9 (parameters: --min-count 2, --k-list 21,41,61,81,99, --min-contig-len 200*).^71^ Open reading frames (ORFs) were predicted from the metagenome assemblies using Prodigal^72^ and a non-redundant ORF set was generated by merging all predicted ORFs and dereplicating using the SeqKit v2.8.2.^73^ A first clustering step was conducted by clustering the non-redundant ORF set with MMseqs2 v13.45111^74^ with the following parameters: –min-seq-id 0.95, -coverage 0.95, --cov-mode 1 and --cluster-mode 2. The sequences extracted were then pooled, and a second round of clustering was executed to generate the final ORF catalog. Next, the high-quality reads from each sample were mapped to the ORF catalog using bowtie2 v2.4.5^75^ and Samtools coverage v1.14^76^ to compute the number of reads mapped.

A matrix based on the normalization of counts using RPKM-SCMG (Reads Per Kilobase of transcript per Million reads mapped and single copy marker gene) was computed by dividing the number of mapped reads by the ORF length, by 10^6^ and by the median count value of ORFs annotated as 10 universal single-copy phylogenetic marker genes (K06942, K01889, K01887, K01875, K01883, K01869, K01873, K01409, K03106, and K03110).^77^ The KEGG Orthology annotation was performed using KofamScan v1.3.0.^78^ Therefore, the normalized ORF counts represent the average number of copies per cell.

To make results across the different types of environments sampled fully comparable, we also generated rarefaction curves based on the sequencing depths attained (Figures S1 and S2).

#### Functional annotation of genes

The ORF catalog was annotated with eggNOG mapper v2.1.5^79^ to retrieve the Clusters of Orthologous Groups of proteins (COGs). The rarefaction curve of the COGs (Figure S2) indicates that the ORF catalog covers most of the COG diversity for the three different environments (water, sediment, and wastewater treatment plant (WWTP)).

#### Antibiotic resistance gene (ARG) screening using multiple tools

The screening for antibiotic resistance genes (ARGs) and the following analyses were conducted over the ORF catalog. In order to achieve a robust and consistent ARG annotation, we conducted a multi-tool ARG screening using 5 ARG screening tools and a total of 9 combinations of the ARG tool databases with two different methodological approaches: Sequence alignment [AMRFinderplus-AMRFinderplusdb,^80^ ABRicate (https://github.com/tseemann/ABRicate) with multiple databases, i.e., NCBI, MEGARes, ARG-ANNOT, CARD and ResFinder), RGI-CARD^81^ and staramr-ResFinder^82^] and Deep learning [DeepARG-DeepARGDB^83^]. More information on each specific tool and database is provided in Section 1.1 of the Supplementary Material.

Potential ARGs detected with sequence alignment and deep learning tools were included in the analysis if their identity and coverage values when aligned to the query sequence exceeded 80%.^23^^,^^54^^,^^83^^,^^84^ In this manner, matches with low similarity were discarded from the analysis, while ARG sequence variability was considered to detect ARG variants in the environment that may have diverged from the collection of clinical ARGs in databases.

Based on the number of ARG hits annotated by just one tool or more than one tool (Figure S3), only those genes annotated by at least two out of the five AMR tools (DeepARG, AMRFinderplus, RGI, Staramr and ABRICATE, considering all databases) were considered ARGs and were included in further analysis. Finally, the annotation was standardized based on the CARD nomenclature.^26^ If two tools reported different ARG classes for a given ORF, the resulting annotation was defined as “ambiguous”. However, if one of the annotations belonged to “unclassified” or “multidrug” classes, while the other annotation/s agreed on a specific AMR class, then the precise annotation was assigned (see Section 1.2 of the Supplementary Material).

#### Steps followed to homogenize the ARG nomenclature

We renamed those ARG class entries that corresponded to the same drug class but were written differently (e.g., “Aminoglycosides” and “aminoglycoside”, “Multi-drug_resistance” and “multidrug” or “mls” and “lincosamide/macrolide/streptogramin”

To assign the annotation, we considered all the databases used by the tools in this study (CARD, KARGVAdefault, AMRFinderplusdb, MEGARes, ARG-ANNOT, NCBI, ResFinder and DeepARGDB).

The predicted ARGs were annotated based on the following criteria.

• If one annotation was dominant compared to the others we used the most prevalent AMR class to classify the gene (e.g., Tetracyclines has an absolute frequency of four and Aminoglycosides of two, we assign the AMR hit as tetracycline).
• In the case, two different annotations were both the two most prevalent ones:
  ○ If the most prevalent labels were {*two different arg_classes* different from *multidrug or unclassified*} then we added the label “*ambiguous annotation”*.
  ○ If the most prevalent labels were {*multidrug* and another *arg_class* (which is not *multidrug*)} then we added the label of the *arg_class*.
  ○ If the most prevalent labels were {*multidrug* and two other *arg_classes*} then we added the label “*multidrug*”.
  ○ If the most prevalent labels were {*multidrug* and *unclassified*} then we added the label as “*unclassified”*.
  ○ If the most prevalent labels were {*unclassified* and another *arg_class* (which is not *multidrug*)} then we added the label of the *arg_class*.

In addition, the following replacements in annotations were conducted according to the CARD database (Alcock, B. P. et al., 2023. CARD 2023: expanded curation, support for machine learning, and resistome prediction at the Comprehensive Antibiotic Resistance Database. Nucleic acids research, 51(D1), D690-D699.).

**Table:** 

Old label	New label
Aminoglycosides	aminoglycoside
Tetracyclines	tetracycline
aminoglycoside:aminocoumarin	aminocoumarin
Aminocoumarins	aminocoumarin
Lipopeptides	lipopeptide
Multi-drug_resistance	multidrug
Sulfonamides	sulfonamide
Tetracyclines	tetracycline
betalactams	beta-lactam
macrolide	MLS
LINCOSAMIDE/MACROLIDE/STREPTOGRAMIN	MLS
LINCOSAMIDE;MACROLIDE;STREPTOGRAMIN	MLS
cationic_antimicrobial_peptides	peptide
metronidazole	nitroimidazole
chloramphenicol	phenicol
rifampin	rifamycin
fosfomycin	phosphonic acid
streptomycin	aminoglycoside
bacitracin	peptide
lincosamide	MLS
streptothricin	nucleoside
colistin	peptide
thiopeptides	peptide
elfamycins	elfamycin
fluoroquinolones	fluoroquinolone
fosmidomycin	phosphonic acid
fusidic_acid	fusidane

#### Taxonomic annotation of genes

For the taxonomic classification of all ARGs in the gene catalog we used Kraken version 2.1.3 Copyright 2013–2023 (https://ccb.jhu.edu/software/kraken2/) with the core_nt Database (https://benlangmead.github.io/aws-indexes/k2). Only classified “C” sequences were used for further analysis with the aim of linking the predicted taxa with the ARGs at the ORF level.

#### MGE sequence annotation

For the annotation of mobile genetic elements, i.e., plasmid and viral sequences, from the ORF non-redundant dataset, we used geNomad v1.8.1^85^ with the following parameters: genomad end-to-end --cleanup --splits 8 genomad_db/--threads 32 --verbose.

### Quantification and statistical analyses

Statistical differences in RPKM counts of AMR classes among environments were assessed using the Kruskal-Wallis test, followed by Dunn’s post hoc test for pairwise multiple comparisons with Bonferroni-adjusted *p*-values using the rstatix package (version 0.7.2) in R. Further details of the statistical test results are provided in Tables S4 and S5 of the supplemental information.
