# Supplementary material for: Diversity of antibiotic resistance genes increases in urbanized lakes: A multi-tool screening
Source: iScience. 2026 Apr 27;29(6):115892. doi: 10.1016/j.isci.2026.115892 (PMC13197638; doi:10.1016/j.isci.2026.115892)
Supplement: Document S1. Figures S1–S4 and Tables S1–S8 [file mmc1.pdf]

iScience, Volume *29*

Supplemental information

Diversity of antibiotic resistance genes increases in urbanized lakes: A multi-tool screening

Pau De Yebra, Luca Zoccarato, John A. Galindo, Daniela Numberger, Nafi'u Abdulkadir, Hans-Peter Grossart, and Alex D. Greenwood

Supplemental Items

Supplementary figures

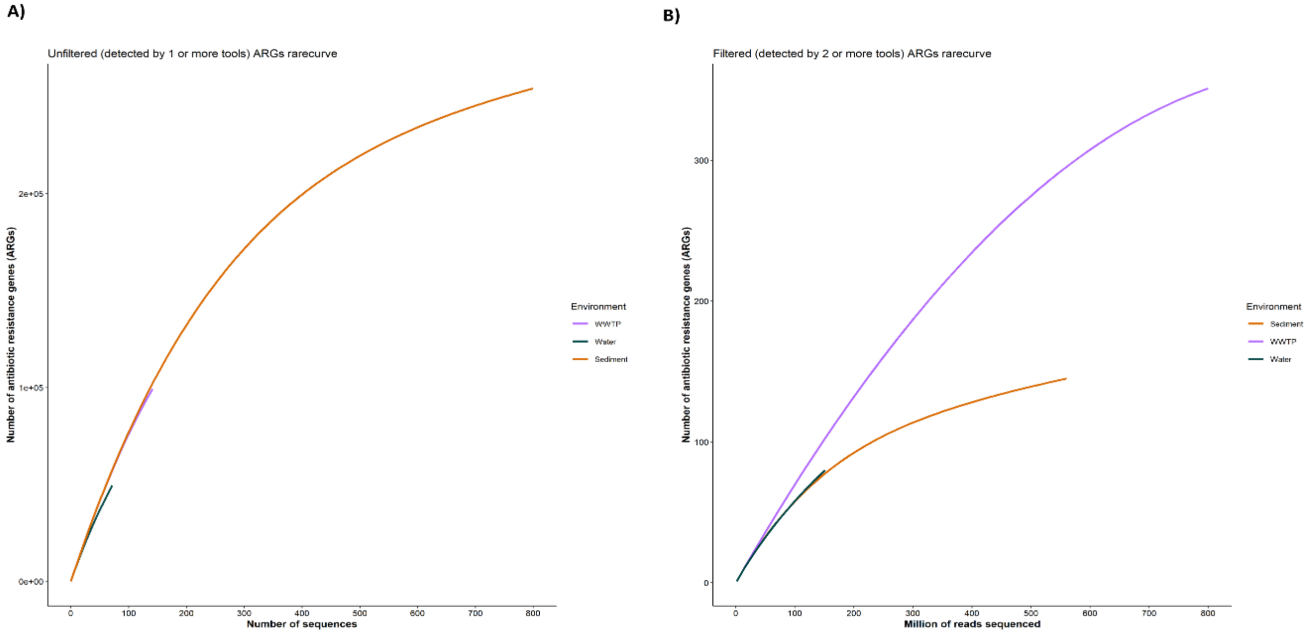

Fig. S1.- Rarefaction curves for the coverage ARGs in relationship to the sequencing depth. After annotating the ORFs for ARGs, each ARG was assigned to one or more environments. Rarefaction curves were computed by grouping the ARGs into three sample types: water, sediments and

WWTP. Panel A) uses as input the unfiltered ARG dataset (all the ARGs predicted by at least 1 screening tools) and B) uses as input the filtered dataset (only those ARGs predicted by at least 2 screening tools). The rarefaction was computed using the function rarecurve from the package *vegan* version 2.6-6.1

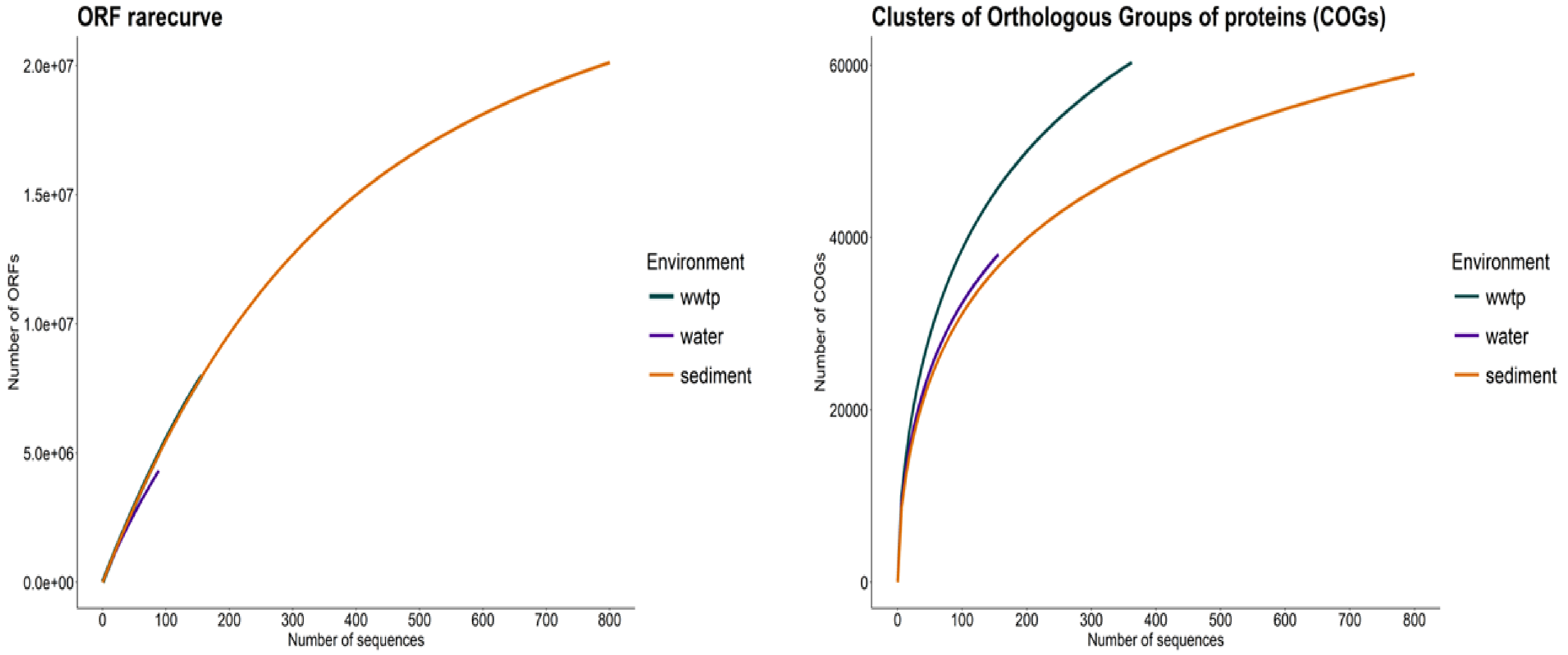

Fig. S2.- Rarefaction curves for the coverage of (A) ORFs and (B) Clusters of Orthologous Groups of proteins (COGs) in relationship to the sequencing depth. ORFs were annotated for COGs using eggnog mapper v2.1.5. Rarefaction curves were computed by grouping the ORFs and COGs

into three sample types: water, sediments and WWTP. The rarefaction was computed using the function rarecurve from the package v*egan* version 2.6-6.1

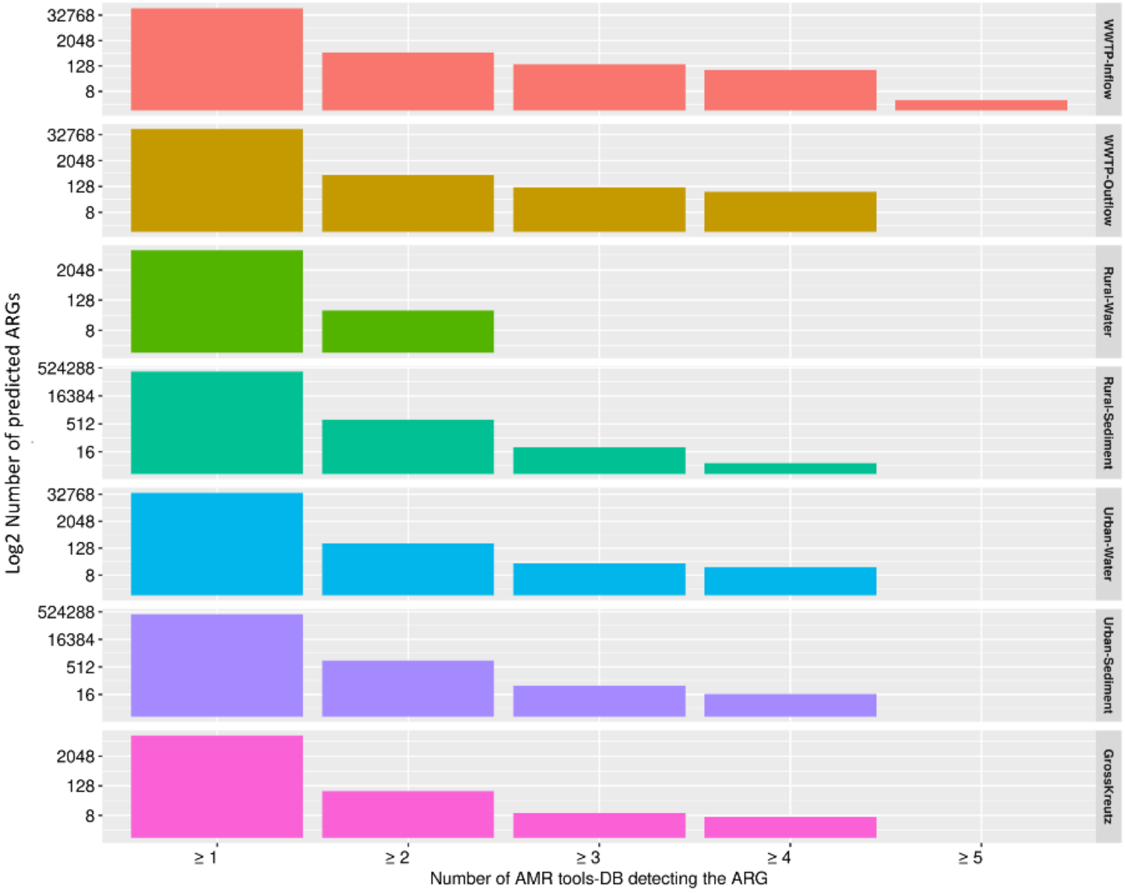

Fig. S3. - Number of ARGs hits per environment that were predicted by the multi-tool approach based on the number of ARGs screening tools that detected each ARG. ORFs were annotated ARGs using 5 ARG tools and then merged together. Then each ARG was grouped depending

on how ARG tools annotated the ARG. The first column corresponds to those ARGs detected by at least one tool whereas the last column contains those ARGs detected by all 5 tools.

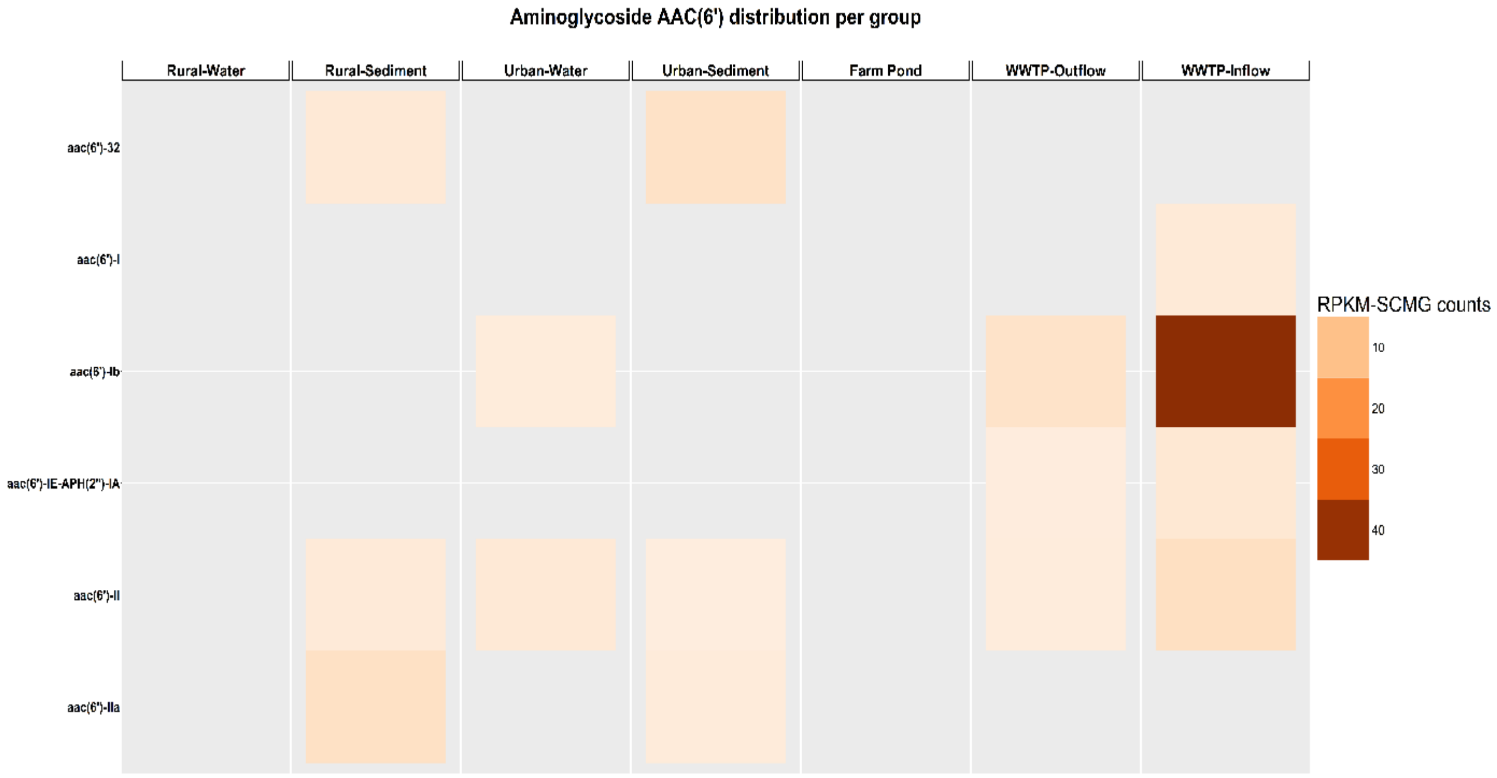

Fig. S4.- Distribution of aminoglycoside gene family AAC(6') across the different environments. Open reading frames (ORFs) were annotated for antibiotic resistance genes (ARGs), filtered by aminoglycoside class and grouped in aminoglycoside gene families according to CARD database (Alcock et al., 2023). Only those genes belonging to the aminoglycoside family AAC(6') were selected. The mean rank of the reads per kilobase per

million mapped reads normalized by single copy marker genes (RPKM-SCMG) counts was computed by mapping the high-quality reads to the ORFs. Each square in the heatmap corresponds to mean rank RPKM-SCMG counts associated to an aminoglycoside AAC(6') gene family and a particular environment.

Supplementary tables

Table S1. Sampling sites and their characteristics. Samples are grouped in four groups: Wastewater treatment plant (WWTP) inflow and outflow; Urban waters which include the lakes, 'Lake Feldberger Haussee', 'Lake Müggelsee' and 'Lake Weißer See'; Rural lakes which include 'Lake Stechlin' and 'Lake Dagow'; and farm pond with a water sample from a farm in Groß Kreutz (Brandenburg). The following fields are listed for each sample: Geographical position, Catchment Area, Inhabitants in the catchment area, Maximum depth (for WWTP is the treatment of wastewater per year), Trophy status and cited References. The exact location of the sampled WWTP cannot be disclosed due to a confidentiality agreement with the WWTP operators.

<table>
<tr><th>Water</th><th>Geographical position</th><th>Catchment Area</th><th>Inhabitants in catchment area</th><th>Max. Depth [m]</th><th>Surface area [km^2]</th><th>Trophy</th><th>Ref</th></tr>
<tr><td>Wastewater Treatment Plant</td><td>Confidential, in agreement with WWTP operators</td><td colspan="2">1.6 million inhabitants (of Berlin)</td><td colspan="2">Treatment of 247,500 m^3 raw wastewater per day</td><td>Hyper-eutrophic</td><td>1</td></tr>
<tr><td>Müggelsee</td><td>52°26'N, 13°39'O</td><td>Berlin Treptow-Köpenick</td><td>271,153 (1,610/km^2)</td><td>8</td><td>7.3</td><td>eutrophic</td><td>2, 3</td></tr>
<tr><td>Weißer See</td><td>52°33'N, 13°27'O</td><td>Berlin Weißensee</td><td>53,737 (6,776/km^2)</td><td>10.6</td><td>0.08</td><td>eutrophic</td><td>4</td></tr>
<tr><td>Feldberger Haussee</td><td>53°20'N, 13°26'O</td><td>Feldberger Seenlandschaft</td><td>4,433 (22/km^2)</td><td>12</td><td>1.3</td><td>eutrophic</td><td>5, 6</td></tr>
<tr><td>Dagowsee</td><td>53°09'N, 13°03'E</td><td rowspan="2">Dagow and Neuglobsow</td><td rowspan="2">383</td><td>9.5</td><td>0.3</td><td>eutrophic</td><td>7–9</td></tr>
<tr><td>Stechlinsee</td><td>53°10'N, 13°02'E</td><td>69.5</td><td>4.3</td><td>oligo-meso-trophic</td><td>10, 11</td></tr>
<tr><td>Großkreutz</td><td>52°23'47.3"N, 12°45'57.9"E</td><td>Potsdam-Mittelmark</td><td>222,570</td><td>-</td><td>-</td><td>-</td><td>-</td></tr>
</table>

Table S2. Comparison of the AMR class prediction (type and number of AMR classes) reported by this and previous studies when using multiple AMR screening tools and databases. Each row corresponds to a single combination of AMR screening tool and database. For each combination the following fields regarding the AMR prediction capacity are listed: AMR tool, Database, Drug classes detected, Number of drug classes detected, Sample type and Reference. The "*" indicates that those predictions showed a very low Balanced Accuracy (BalAcc), near 0.50, resulting in an AMR class detection which is likely to be inaccurate (Marini et al., 2022).

	AMR Tool	Database used	Drug classes detected	Number of drug classes detected	Input sample	Reference
1	**Multi-tool approach** (Same tools from rows 2-6, filtering hits detected by ≥ 2 tools)	Same databases from rows 2-6	Aminoglycosides, Beta-lactams, Cephalosporins, Diaminopyrimidine, Fluoroquinolone, Glycopeptide, MLS, Multidrug, Nitromidazole, Nucleoside, Peptide, Phenicol, Phosphonic acid, Rifamycin, Sulfonamide and Tetracycline. Additionally drug resistance against Biocides and Metals.	18	Detection in at least one environment amongst WWTP inflow, WWTP outflow, farm pond or freshwater lakes (water or sediments)	This study
1	**ABRICATE**	ResFinder DB, NCBI, ARG-ANNOT, CARD and MEGARes	Aminoglycosides, Beta-lactams, Cephalosporins, Diaminopyrimidine, Fluoroquinolone, Glycopeptide, MLS, Multidrug, Nitromidazole, Nucleoside, Peptide, Phenicol, Phosphonic acid, Rifamycin, Sulfonamide and Tetracycline. Additionally drug resistance against Biocides.	18	Detection in at least one environment amongst WWTP inflow, WWTP outflow, farm pond or freshwater lakes (water or sediments)	This study
2	**RGI**	CARD	Aminoglycosides, Beta-lactams, Cephalosporins, Diaminopyrimidine, Fluoroquinolone, Glycopeptide, MLS, Multidrug, Nitromidazole, Nucleoside, Peptide, Phenicol, Phosphonic acid, Rifamycin, Sulfonamide and Tetracycline. Additionally drug resistance against Biocides.	17	Detection in at least one environment amongst WWTP inflow, WWTP outflow, farm pond or freshwater lakes (water or sediments)	This study
3	**AMRfinderplus**	AMRfinderplus DB	Aminoglycosides, Beta-lactams, Cephalosporins, Diaminopyrimidine, Fluoroquinolone, Glycopeptide, MLS, Nitromidazole, Nucleoside, Peptide, Phenicol, Phosphonic acid, Rifamycin, Sulfonamide and Tetracycline.	16	Detection in at least one environment amongst WWTP inflow, WWTP outflow, farm pond or freshwater lakes (water or sediments)	This study

			Additionally drug resistance against Biocides.			
4	**DeepARG**	AMRfinderplus DB	Aminoglycosides, Beta-lactams, Diaminopyrimidine, Fluoroquinolone, Glycopeptide, MLS, Multidrug, Nucleoside, Peptide, Phenicol, Phosphonic acid, Rifamycin, Sulfonamide and Tetracycline. Additionally drug resistance against Aminocoumarin.	15	Detection in at least one environment amongst WWTP inflow, WWTP outflow, farm pond or freshwater lakes (water or sediments)	This study
5	**Staramr**	ResFinder DB	Aminoglycosides, Beta-lactams, Diaminopyrimidine, Fluoroquinolone, MLS, Nitromidazole, Peptide, Phenicol, Phosphonic acid, Rifamycin, Sulfonamide and Tetracycline.	12	Detection in at least one environment amongst WWTP inflow, WWTP outflow, farm pond or freshwater lakes (water or sediments)	This study
6	**ResFinder v2.1 (web tool)**	not specified	Aminoglycosides, Beta-lactams, Cephalosporins, Folate Pathway Inhibitors (Sulfonamides and Diaminopyrimidines) and Tetracycline	6	Salmonella strains collected from Broiler Chickens	Cooper et al., 2020
7	**KMA v1.17**	ResFinder, NCBI	Aminoglycosides, Beta-lactams, Cephalosporins, Folate Pathway Inhibitors (Sulfonamides and Diaminopyrimidines) and Tetracycline	6	Salmonella strains collected from Broiler Chickens	Cooper et al., 2021
8	**SRST2**	ResFinder, ARG-Annot, NCBI	Aminoglycosides, Beta-lactams, Cephalosporins, Penicillins and Tetracycline	5	Salmonella strains collected from Broiler Chickens	Cooper et al., 2022
9	**RGI**	CARD	Aminoglycosides, Beta-lactams, Cephalosporins, Penicillins and Tetracycline	5	Salmonella strains collected from Broiler Chickens	Cooper et al., 2023
10	**ABRICATE**	ResFinder DB, NCBI, ARG-ANNOT, CARD and MEGARes	Aminoglycosides, Beta-lactams, Fluoroquinolone, MLS, Efflux pump transporter (not an CARD drug class) and Tetracycline	6	Multiple sources (sewage, monkey, human, potable water and chicken)	Gomes et al., 2023
11	**RGI**	CARD	Aminoglycosides, Beta-lactams, Fluoroquinolone, MLS, Efflux pump transporter (not an ARO class) and Tetracycline	6	Multiple sources (sewage, monkey, human, potable water and chicken)	Gomes et al., 2023

12	**AMRPlusPlus**	MEGARes	Aminoglycosides*, Beta-lactams, Fluoroquinolone, MLS*, Phenicols*, Tetracycline and Diaminopyrimidines	4 accurately; 3 low precission*	500 isolates according to the following criteria: sequenced using Illumina platform; available AMR resistance profiles determined by phenotypic AST; available NCBI BioProject; and other parameters stated in the publication	Marini et al., 2022
13	**Meta-MARC**	MEGARes	Aminoglycosides*, Beta-lactams, Fluoroquinolone*, MLS*, Phenicols*, Tetracycline and Diaminopyrimidines*	2 accurately; 5 low precission*	500 isolates according to the following criteria: sequenced using Illumina platform; available AMR resistance profiles determined by phenotypic AST; available NCBI BioProject; and other parameters stated in the publication	Marini et al., 2022
14	**KARGA**	MEGARes, or any other fasta AMR DB	Aminoglycosides, Beta-lactams, Fluoroquinolone, MLS, Phenicols, Tetracycline and Diaminopyrimidines	7 accurately	500 isolates according to the following criteria: sequenced using Illumina platform; available AMR resistance profiles determined by phenotypic AST; available NCBI BioProject; and other parameters stated in the publication	Marini et al., 2022
15	**DeepARG**	DeepARG-DB	Aminoglycosides*, Beta-lactams, Fluoroquinolone*, MLS, Phenicols* and Tetracycline.	2 accurately; 5 low precission*	500 isolates according to the following criteria: sequenced using Illumina platform; available AMR resistance profiles determined by phenotypic AST; available NCBI BioProject; and other parameters stated in the publication	Marini et al., 2022
16	**ResFinder**	ResFinder DB , PointFinder DB	Aminoglycosides, Beta-lactams*, Fluoroquinolone, MLS, Phenicols, Tetracycline and Diaminopyrimidines	6 accurately; 1 low precission*	500 isolates according to the following criteria: sequenced using Illumina platform; available AMR resistance profiles determined by phenotypic AST; available NCBI BioProject; and other parameters stated in the publication	Marini et al., 2022
17	**Staramr**	ResFinder DB, PointFinder DB, PlasmidFinder DB	Aminoglycosides, Cephalosporin, Diaminopyrimidine, Phosphonic acid antibiotic, MLS, Nitrofuran, Penicillin, Phenicol, Fluoroquinolone, Sulfonamides, Tetracycline and Trimethoprim.	12	Simulated highly and low resistant mock community based on sequenced strains with known phenotypes. Following the requisites: (1) the strain had extensive antibiotic susceptibility CLSI or EUCAST testing data, (2) the strain was isolated from human tissue, (3) the strain was the cause of a clinical infection, (4) the FASTA was available to download from NCBI BioSample Database.	Wissel A et al., 2023

18	**Srax**	CARD by default	Aminoglycosides, Beta-lactams, Diaminopyrimidine, MLS, Oxazolidinone, Fluoroquinolone.	6	Simulated highly and low resistant mock community based on sequenced strains with known phenotypes. Following the requisites: (1) the strain had extensive antibiotic susceptibility CLSI or EUCAST testing data, (2) the strain was isolated from human tissue, (3) the strain was the cause of a clinical infection, (4) the FASTA was available to download from NCBI BioSample Database.	Wissel A et al., 2024
19	**Shortbread**	AMR gene marker database from 849 AR protein families from the ARDB19 and independent curation	Aminoglycosides, Beta-lactams, MLS, Fluoroquinolone and Tetracycline.	5	Simulated highly and low resistant mock community based on sequenced strains with known phenotypes. Following the requisites: (1) the strain had extensive antibiotic susceptibility CLSI or EUCAST testing data, (2) the strain was isolated from human tissue, (3) the strain was the cause of a clinical infection, (4) the FASTA was available to download from NCBI BioSample Database.	Wissel A et al., 2025
20	**RGI**	CARD	Aminoglycosides, Beta-lactams, Glycopeptide, Cephalosporin, Diaminopyrimidine, Phosphonic acid antibiotic, Antibacterial free fatty acids, Glycopeptide, MLS, Nitrofuran, Nitroimidazole, Nucleoside, Oxazolidinone, Peptide, Phenicol, Pleuromutilin, Fluoroquinolone, Rhodamine (not an CARD drug class), Rifamycin, Sulfonamides and Tetracycline. Additionally drug resistance against Biocides.	22	Simulated highly and low resistant mock community based on sequenced strains with known phenotypes. Following the requisites: (1) the strain had extensive antibiotic susceptibility CLSI or EUCAST testing data, (2) the strain was isolated from human tissue, (3) the strain was the cause of a clinical infection, (4) the FASTA was available to download from NCBI BioSample Database.	Wissel A et al., 2026
21	**Resfinder 4**	ResFinder 4 DB	Aminoglycosides, Beta-lactams, Glycopeptide, Diaminopyrimidine, Phosphonic acid antibiotic, MLS, Nitrofuran, Phenicol, Fluoroquinolone, Sulfonamides, Tetracycline and Trimethoprim. Additionally drug resistance against Biocides.	13	Simulated highly and low resistant mock community based on sequenced strains with known phenotypes. Following the requisites: (1) the strain had extensive antibiotic susceptibility CLSI or EUCAST testing data, (2) the strain was isolated from human tissue, (3) the strain was the cause of a clinical infection, (4) the FASTA was available to download from NCBI BioSample Database.	Wissel A et al., 2027

22	**fARGene**	Hidden Markov models for quinolone, tetracycline, and beta lactamases	Beta-lactams, Fluoroquinolone, Tetracycline and Trimethoprim.	4	Simulated highly and low resistant mock community based on sequenced strains with known phenotypes. Following the requisites: (1) the strain had extensive antibiotic susceptibility CLSI or EUCAST testing data, (2) the strain was isolated from human tissue, (3) the strain was the cause of a clinical infection, (4) the FASTA was available to download from NCBI BioSample Database.	Wissel A et al., 2028
23	**DeepARG**	DeepARG-DB	Aminoglycosides, Beta-lactams, Glycopeptide, Cephalosporin, Diaminopyrimidine, Phosphonic acid antibiotic, Glycopeptide, MLS, Nitroimidazole, Peptide, Phenicol, Fluoroquinolone, Sulfonamides, Tetracycline. Additionally drug resistance against Biocides.	15	Simulated highly and low resistant mock community based on sequenced strains with known phenotypes. Following the requisites: (1) the strain had extensive antibiotic susceptibility CLSI or EUCAST testing data, (2) the strain was isolated from human tissue, (3) the strain was the cause of a clinical infection, (4) the FASTA was available to download from NCBI BioSample Database.	Wissel A et al., 2029
24	**AMRfinderplus**	AMRfinderplus DB	Aminoglycosides, Beta-lactams, Glycopeptide, Cephalosporin, Diaminopyrimidine, Phosphonic acid antibiotic, Antibacterial free fatty acids, MLS, Nitrofuran, Nucleoside, Peptide, Phenicol, Fluoroquinolone, Sulfonamides, Tetracycline and Trimethoprim. Additionally drug resistance against Biocides and Metals.	18	Simulated highly and low resistant mock community based on sequenced strains with known phenotypes. Following the requisites: (1) the strain had extensive antibiotic susceptibility CLSI or EUCAST testing data, (2) the strain was isolated from human tissue, (3) the strain was the cause of a clinical infection, (4) the FASTA was available to download from NCBI BioSample Database.	Wissel A et al., 2030
25	**ABRICATE**	ResFinder DB, NCBI, ARG-ANNOT, CARD and MEGARes	Aminoglycosides, Beta-lactams, Cephalosporin, Diaminopyrimidine, Phosphonic acid antibiotic, Glycopeptide, MLS, Phenicol, Fluoroquinolone, Sulfonamides, Tetracycline and Trimethoprim.	12	Simulated highly and low resistant mock community based on sequenced strains with known phenotypes. Following the requisites: (1) the strain had extensive antibiotic susceptibility CLSI or EUCAST testing data, (2) the strain was isolated from human tissue, (3) the strain was the cause of a clinical infection, (4) the FASTA was available to download from NCBI BioSample Database.	Wissel A et al., 2031

Table S3. Number of ARG hits detected for each intersection of environment (higher number of shared ARGs). Open reading frames (ORFs) were annotated for antibiotic resistance genes (ARGs) and only those present in more than one environment were selected. Then the number of ARG hits for each intersection was listed in decreasing order. Intersecting environments with only 1 ARG are shown in Table S5.

Intersections between environments	Number of ARG hits
WWTP inflow and outflow	144
WWTP inflow	70
WWTP inflow and outflow and urban water	23
Urban and rural sediments	21
WWTP inflow and outflow and urban sediment	16
WWTP inflow and outflow and farm pond	7
WWTP inflow and outflow, urban water and urban sediment	6
WWTP inflow and outflow, rural and urban sediment	5
WWTP inflow and outflow and rural sediment	5
WWTP inflow and outflow, farm pond and urban water	5
WWTP inflow and urban sediment	2
WWTP inflow and farm pond	2
Rural sediment	2

Table S4. Kruskal-Wallis statistics for those AMR classes which showed significant differences in the mean rank of the reads per kilobase per million mapped reads normalized by single copy marker genes (RPKM-SCMG) when compared between environments. This table supports boxplots of Fig.3 (meaning a p-value adjusted > 0.05). Open reading frames (ORFs) were annotated for antibiotic resistance genes (ARGs) and the mean rank of the RPKM counts was computed by mapping the high-quality reads to the ORFs. This table also includes those AMR classes that show a non-adjusted p-value < 0.05. P-value adjusted significance {* < 0.05, ** < 0.01, *** < 0.001, **** < 0.0001}.

**ARG_class**	**.y.**	**n**	**statistic**	**df**	**p**	**method**	**p.adj**	**p.adj.signif**
aminoglycoside	count	177	49.63	5	1.64e-09	Kruskal-Wallis	3.77e-08	****
beta-lactam	count	137	34.99	4	4.66e-07	Kruskal-Wallis	1.07e-05	****
drug_biocide_resistance	count	127	57.39	5	4.20e-11	Kruskal-Wallis	9.66e-10	****
mls	count	81	24.58	4	6.09e-05	Kruskal-Wallis	1.40e-03	**
tetracycline	count	52	33.54	5	2.93e-06	Kruskal-Wallis	6.73e-05	****

Table S5. Dunn's test statistics for the pairwise mean rank comparison of those AMR classes which showed significant differences in the mean rank of the reads per kilobase per million mapped reads normalized by single copy marker genes (RPKM-SCMG) when compared between environments. This table supports boxplots of Fig.3 (meaning a p-value adjusted > 0.05). Open reading frames (ORFs) were annotated for antibiotic resistance genes (ARGs) and the mean rank of the RPKM counts was computed by mapping the high-quality reads to the ORFs. This table also includes those AMR classes that show a non-adjusted p-value < 0.05. P-value adjusted significance {* < 0.05, ** < 0.01, *** < 0.001, **** < 0.0001}.

ARG_class	.y.	group1	group2	n1	n2	statistic	p	p.adj	p.adj.signif
aminoglycoside	count	WWTP-Inflow	Farm-Pond	36	5	-4.91	8.82e-07	5.73e-05	****
aminoglycoside	count	WWTP-Inflow	Urban-Sediment	36	55	-5.24	1.59e-06	1.03e-04	****
aminoglycoside	count	WWTP-Inflow	Urban-Water	36	36	-4.56	4.96e-06	3.22e-04	***
aminoglycoside	count	WWTP-Outflow	Farm-Pond	30	5	-3.42	6.24e-04	4.03e-02	*
aminoglycoside	count	Farm-Pond	Rural-Sediment	5	18	4.35	5.42e-04	3.52e-02	*
beta-lactam	count	WWTP-Inflow	Urban-Sediment	54	19	-4.72	2.35e-06	1.53e-04	***
beta-lactam	count	WWTP-Inflow	Rural-Sediment	54	19	-3.92	8.61e-05	5.60e-03	**
beta-lactam	count	WWTP-Outflow	Urban-Sediment	49	19	-4.02	5.69e-05	3.70e-03	**

ARG_class	.y.	group1	group2	n1	n2	statistic	p	p.adj	p.adj.signif
beta-lactam	count	WWTP-Outflow	Rural-Sediment	49	19	-3.37	7.26e-04	4.72e-02	*
drug_biocide_resistance	count	WWTP-Outflow	WWTP-Inflow	61	61	-4.36	1.67e-05	1.19e-03	***
drug_biocide_resistance	count	Farm-Pond	Rural-Sediment	5	18	-3.99	6.53e-02	4.24e-04	**
drug_biocide_resistance	count	Urban-Sediment	Rural-Sediment	61	18	-4.93	7.96e-06	5.17e-04	****
drug_biocide_resistance	count	WWTP-Inflow	Urban-Water	61	61	-4.13	3.49e-04	2.26e-02	**
tetracycline	count	WWTP-Inflow	Farm-Pond	36	5	-3.55	3.83e-04	2.49e-02	*
tetracycline	count	WWTP-Inflow	Urban-Sediment	18	18	-4.05	4.92e-06	3.19e-04	**
tetracycline	count	WWTP-Inflow	Rural-Sediment	18	18	-3.50	4.49e-04	2.91e-02	*

Table S6. ARG intersection for those environments which share only one antibiotic resistance gene (ARG). Open reading frames (ORFs) were annotated for antibiotic resistance genes (ARGs) and only those combinations of environments which shared 1 ARG were selected. Then the number of ARG hits for each intersection was listed.

**Intersections between environments**	**Number of ARG hits**
Rural sediment, urban water and urban sediment	1
WWTP outflow	1
WWTP outflow and urban sediment	1
WWTP outflow and urban sediment and rural sediments	1
WWTP inflow and rural sediment	1
WWTP inflow, urban water and rural sediment	1
WWTP inflow and outflow, farm pond and rural sediment	1
WWTP inflow and outflow, rural sediment and urban water	1
WWTP inflow and outflow, urban sediment and farm pond	1
WWTP inflow and outflow, urban sediment, urban water, rural sediment	1
WWTP inflow and outflow, urban sediment, urban water, rural sediment and farm pond	1

Table S7. AMR class abundance comparison between the WWTP Inflow and the WWTP Outflow. Open reading frames (ORFs) were annotated for antibiotic resistance genes (ARGs) and only those belonging to the WWTP inflow and WWTP outflow and, which had an AMR class assigned, were listed and sorted by abundance (RPKM-SCMG counts).

group	ARG_class	counts			group	ARG_class	counts
WWTP-Inflow	sulfonamide	62.11926176		1	WWTP-Outflow	sulfonamide	51.46090567
WWTP-Inflow	tetracycline	43.29441272		2	WWTP-Outflow	MLS	9.313243386
WWTP-Inflow	MLS	18.6230147		3	WWTP-Outflow	beta-lactam	8.913506546
WWTP-Inflow	cephalosporin	17.02206724		4	WWTP-Outflow	tetracycline	8.806977195
WWTP-Inflow	aminoglycoside	13.60019685		5	WWTP-Outflow	aminoglycoside	5.835370769
WWTP-Inflow	beta-lactam	11.10864217		6	WWTP-Outflow	diaminopyrimidine	3.417023701
WWTP-Inflow	phenicol	10.97504052		7	WWTP-Outflow	multidrug	3.065306204
WWTP-Inflow	fluoroquinolone	9.853703689		8	WWTP-Outflow	fluoroquinolone	2.219676743
WWTP-Inflow	diaminopyrimidine	7.6070189		9	WWTP-Outflow	cephalosporin	2.196473552
WWTP-Inflow	peptide	3.442193257		10	WWTP-Outflow	phenicol	2.028349205
WWTP-Inflow	multidrug	3.248614903		11	WWTP-Outflow	peptide	1.55388725
WWTP-Inflow	biocide resistance	3.220638809		12	WWTP-Outflow	biocide resistance	1.14984574
WWTP-Inflow	rifamycin	2.865307999		13	WWTP-Outflow	phosphonic acid	1.14088951
WWTP-Inflow	phosphonic acid	2.421449837		14	WWTP-Outflow	rifamycin	1.082781457
WWTP-Inflow	nitroimidazole	2.248053219		15	WWTP-Outflow	biocide and metal resistance	0.747727273
WWTP-Inflow	biocide and metal resistance	1.406362499		16	WWTP-Outflow	nucleoside	0.622857143

WWTP-Inflow	glycopeptide	1.141990919					
WWTP-Inflow	nucleoside	0.659293726					

Table S8. Single-tool detection profile of the 5 ARG screening tools used in the multi-tool approach. The following fields are listed for each tool: Total number of ARG hits detected, followed by the number of ARG hits detected in each drug class in decreasing order. Highlighted in bold are those classes that appear to be unique in each tool.

	**ABRICATE**		**RGI**		**AMRfinderplus**		**DeepARG**		**Staramr**	
**Total 1-hits detected**		**117**		**0**		**33**		**415**		**0**
	beta-lactam	39		**NA**	beta-lactam	20	**peptide**	202		**NA**
	drug_biocide_resistance	33		**NA**	mls	10	multidrug	121		**NA**
	**drug_biocide_metal_resistance**	13		**NA**	drug_biocide_resistance	2	unclassified	20		**NA**
	glycopeptide	12		**NA**	phosphonic acid	1	**aminoglycoside**	22		**NA**
	unclassified	6		**NA**			mls	13		**NA**
	mls	4		**NA**			fluoroquinolone	11		**NA**
	multidrug	4		**NA**			**aminocoumarin**	10		**NA**
	fluoroquinolone	2		**NA**			beta-lactam	10		**NA**
	tetracycline	2		**NA**			glycopeptide	3		**NA**
	**nitroimidazole**	1		**NA**			phosphonic acid	2		**NA**
	**phenicol**	1		**NA**			tetracycline	1		**NA**
